# Recognition of 8-Oxo-2′-deoxyguanosine in DNA Using the Triphosphate of 2′-Deoxycytidine Connecting the 1,3-Diazaphenoxazine Unit, dCdapTP

**DOI:** 10.3390/molecules29102270

**Published:** 2024-05-11

**Authors:** Takato Sakurada, Yuta Chikada, Ryo Miyahara, Yosuke Taniguchi

**Affiliations:** 1 Graduate School of Pharmaceutical Sciences, Kyushu University, 3-1-1 Maidashi, Higashi-ku, Fukuoka 812-8582, Japan; takatorres.31@gmail.com (T.S.); chikada.yuta.840@s.kyushu-u.ac.jp (Y.C.); c51339rm@gmail.com (R.M.); 2 Faculty of Medicine, Dentistry and Pharmaceutical Sciences, Okayama University, 1-1-1 Tsushima-naka, Kita-ku, Okayama 700-8530, Japan

**Keywords:** 8-oxo-2′-deoxyguanosine, single nucleotide elongation reaction, artificial nucleoside triphosphate, 2′-deoxycytidine derivatives

## Abstract

DNA is constantly damaged by various external and internal factors. In particular, oxidative damage occurs in a steady state, and 8-oxo-2′-deoxyguanosine (oxodG) is known as the main oxidative damage. OxodG is a strong genotoxic nucleoside and is thought to be involved in the pathogenesis of cancer and neurological diseases. However, a breakthrough method to detect the position of oxodG in DNA has not yet been developed. Therefore, we attempted to develop a novel method to detect oxodG in DNA using artificial nucleosides. Recently, we have succeeded in the recognition of oxodG in DNA by a single nucleotide elongation reaction using nucleoside derivatives based on a purine skeleton with a 1,3-diazaphenoxazine unit. In this study, we developed a new nucleoside derivative with a pyrimidine skeleton in order to further improve the recognition ability and enzymatic reaction efficiency. We, therefore, designed and synthesized 2′-deoxycytidine-1,3-diazaphenoxazine (Cdap) and its triphosphate derivatives. The results showed that it was incorporated into the primer strand relative to the dG template because of its cytidine skeleton, but it was more effective at the complementary position of the oxodG template. These results indicate that the new nucleoside derivative can be considered as one of the new candidates for the detection of oxodG in DNA.

## 1. Introduction

DNA and nucleotides in living cells are constantly at risk of damage. This can be caused by external factors, such as ultraviolet radiation, or internal factors, such as reactive oxygen species (ROS), both of which are unavoidable in the process of life. In particular, ROS are very familiar to aerobic organisms because they are generated during immune responses and normal metabolic activities. Therefore, reactive oxygen species remove enzymes, such as superoxide dismutase (SOD), and damage repair mechanisms have been developed. However, a decline in their functions due to illness or mental stress leads to the accumulation of damage, which affects cells, tissues, genes, and other organs at various levels. In general, “the state in which the oxidative capacity of ROS in vivo exceeds its antioxidant capacity” is called oxidative stress [1], and the definition and quantification methods of indicators have been actively studied in relation to diseases and stress. Oxidative stress damages all biomolecules, and DNA damage, in particular, is an extremely important issue because DNA itself has genetic information. DNA oxidative metabolites have also attracted attention as good biomarkers for assessing the burden of oxidative stress on living cells [2]. It has been reported that nucleobases containing purine bases have a low oxidation potential and can easily induce oxidative damage [3,4,5,6,7,8,9]. Among them, 8-oxo-2′-deoxyguanosine (oxodG), in which the 8-position of 2′-deoxyguanosine (dG) is oxidized, has been actively studied because several repair enzymes have been found in vivo, and it is most commonly used as an indicator of nucleobase oxidation. Although oxodG occurs randomly in DNA [10,11], its presence at specific positions is thought to play an important role in biochemistry, which makes its analysis by DNA sequencing increasingly important [12]. However, no simple method has been developed to identify the location of 8-oxo-dG occurrence in DNA using conventional detection methods [13,14,15,16,17,18,19,20,21,22,23].

**Figure 1:**
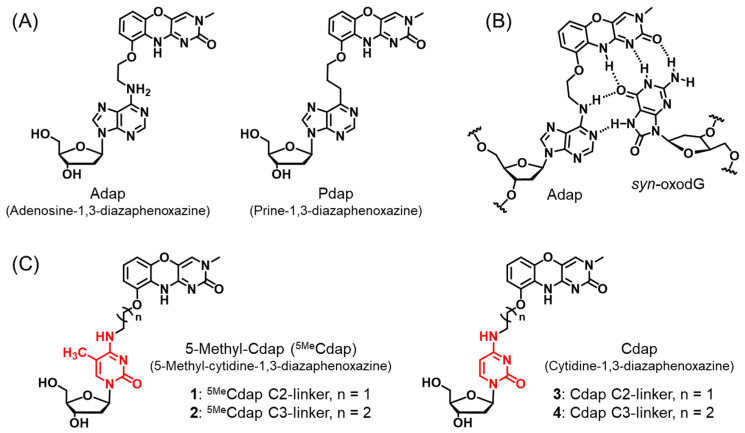
(**A**) Structures of Adap and Pdap; (**B**) speculated recognition structure of Adap to syn-oxodG; (**C**) structures of new Cdap derivatives.

Our group has been investigating the use of artificial nucleoside analogs to directly recognize oxodG in DNA through specific hydrogen bonding formation and to apply them to damaged DNA sequencing methods. Recently, we have succeeded in developing novel nucleoside derivatives, Adenosine-1,3-diazaphenoxazine (Adap) and Purine-1,3-diazaphenoxazine (Pdap) (Figure 1A), consisting of a phenoxazine unit attached to a purine skeleton, which can recognize 8-oxodG in DNA (Figure 1B) [24,25,26]. However, these artificial trinucleotide derivatives have a drawback in that not only the oxodG but also the T of the template strand is incorporated into the primer strand in the single nucleotide insertion reaction using DNA polymerase. We are currently investigating various approaches to overcome this issue, and as one of them, we focused on new derivatives with a pyrimidine skeleton. Therefore, in this paper, based on the previous concept of recognition mode of artificial nucleoside analogs, we investigate the length of the linker that connects the phenoxazine unit from the pyrimidine skeleton to form a hydrogen bonding with oxodG in the *syn*-conformation (Figure 1C). 

## 2. Results and Discussion

### 2.1. Synthesis of 3′-,5′-Diol Compounds

**Scheme 1:**
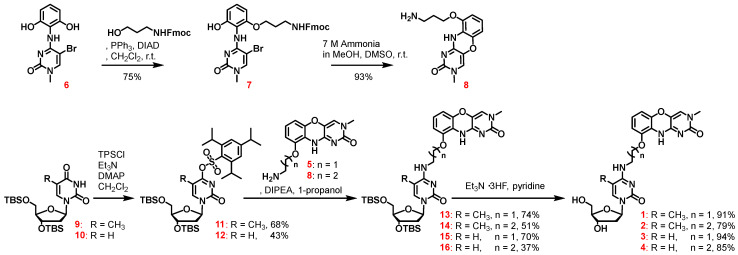
Synthesis of 3′-,5′-diol compounds of Cdap derivatives.

First, we synthesized 1,3-diazaphenoxazine units with a C2-linker or C3-linker (Scheme 1). The C2-linker compound **5** was synthesized according to a previous report [24]. The C3-linker derivative, on the other hand, was synthesized using the Fmoc-protected propanolamine, 3-(Fmoc-amino)-1-propanol, and compound **6** in the Mitsunobu reaction to obtain compound **7**. Immediately after a quick purification process, target compound **8** was successfully obtained in good yield by the cycloaddition reaction and deprotection reaction of the Fmoc group using ammonia methanol. Then, using thymidine or 2′-deoxyuridine as starting materials, TBS protection of the hydroxyl groups (**9** or **10**) and TPS of the carbonyl group at 4-position of the corresponding nucleobase were performed to obtain compounds **11** or **12** [27,28]. Phenoxazine units with different linker lengths were connected to these compounds by the S_N_Ar reaction to obtain compounds **13**–**16**. The TBS groups of the hydroxyl groups of these compounds were deprotected to obtain the corresponding 3′-,5′-diol compounds of Cdap derivatives (**1**–**4**). 

### 2.2. Synthesis of Triphosphate Compounds

**Scheme 2:**
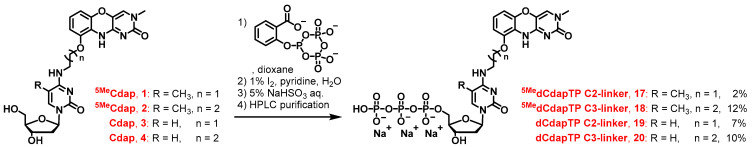
Synthesis of triphosphate compounds of dCdap derivatives.

In this reaction, 2-chloro-4*H*-1,3,2-benzodioxaphosphorin-4-one and tributylammonium pyrophosphate were first mixed to produce a cyclic triphosphate reagent [29]. Then, the hydroxyl group at the 5′-position of the sugar part was modified by mixing the adjusted reagent with the corresponding 3′-,5′-diol compounds. The cyclic triphosphate was oxidized and hydrolyzed to synthesize the desired triphosphate compounds **17**–**20**. Purification was performed by HPLC, and each structure was determined by ^31^P-NMR and high-resolution mass measurements (Scheme 2). For compounds with poor isolated yields, the corresponding 3′-,5′-diol compound was confirmed by HPLC. This may be due to a problem with the reagents or the 3′-,5′-diol compound’s physical properties that prevented the first reaction from completing. However, we did not study the reaction conditions further because we obtained a sufficient amount of trisphosphate compounds to evaluate their functions.

### 2.3. Evaluation of oxodG Recognition Ability by Single Nucleotide Elongation Reaction

**Figure 2:**
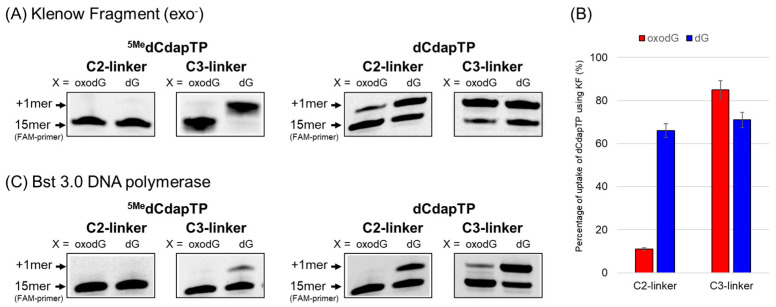
(**A**) Gel results of single nucleotide primer elongation reactions using Klenow Fragment; (**B**) bar graph of the uptake percentage of dCdapTP using KF; and (**C**) gel results of single nucleotide primer elongation reactions using Bst 3.0 DNA polymerase. Conditions: 1.0 µM 15 mer primer and 25 mer template (X) duplex DNA, 0.2 or 0.1 unit/µL of KF or 0.2 unit/µL of Bst 3.0 polymerase in the corresponding reaction buffer, 5 µM of dCdapTP derivatives, and incubation for 10–30 min in a reaction volume of 10 µL.

**Figure 3:**
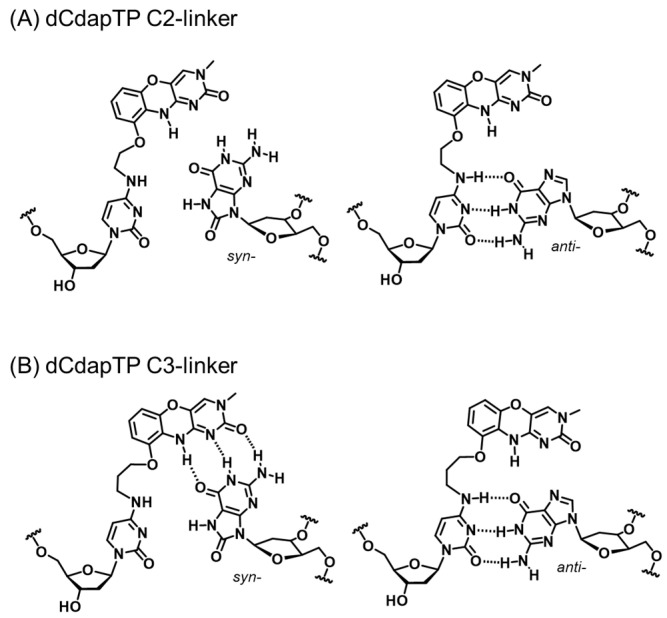
Speculated recognition structures of the dCapTP (**A**) C2-linker and (**B**) C3-linker for *syn*-oxodG and *anti*-dG.

We tested the recognition ability between oxodG and dG in oligonucleotides of new triphosphate derivatives using Klenow Fragment (KF) and Bst 3.0 DNA polymerase. The gel results of the single nucleotide elongation reaction are shown in Figure 2. When using KF, the ^5Me^dCdapTP with a C2-linker was not incorporated into the primer chain. On the other hand, for the ^5Me^dCdapTP with a C3-linker, the elongation reaction proceeded only when the template DNA contained dG (Figure 2A, left). Interestingly, primer elongation reactions were observed for template DNA containing oxodG or dG using dCdapTPs, especially when dCdapTP with a C3-linker was well incorporated into the primer strand opposite the oxo-dG template DNA (Figure 2A, right). Therefore, the uptake percentages were calculated from the fluorescence intensity of these bands (Figure 2B). The value of dCdapTP with a C2-linker was 11% for oxodG template DNA and 66% for dG template DNA. On the other hand, the value of dCdapTP with a C3-linker was 85% for oxodG template DNA and 71% for dG template DNA (Figure 2A, right). These results indicated that the longer linker length may have enabled the artificial nucleotide analog to successfully recognize the *syn*-oriented oxodG (Figure 3). In contrast, the linker length does not seem to affect the *anti*-oriented dG since there is no significant change in the uptake percentage (Figure 3). When using Bst 3.0 DNA polymerase, ^5Me^dCdapTP with a C2- or C3-linker was almost never incorporated into the primer strand (Figure 2C). In the case of dCdapTPs, the single nucleotide elongation reaction was confirmed; however, the results showed that it was well incorporated at the dG template DNA.

### 2.4. Steady-State Kinetic Study of dCapTP Using Klenow Fragment 

In order to investigate the uptake efficiency for dCdapTP using KF in more detail, a steady-state kinetic analysis was carried out (Table 1). In comparing the uptake efficiencies, dCdapTP with a C2-linker is clearly incorporated at the complementary position of dG template DNA (7.22 × 10^6^ %min^−1^M^−1^), followed by the oxo-dG template DNA (2.91 × 10^6^ %min^−1^M^−1^) (Table 1, entries 2 and 1). It is then taken up against dC, T, and dA template DNA (1.13 × 10^6^, 0.88 × 10^6^, and 0.69 × 10^6^ %min^−1^M^−1^, respectively), in that order, but with lower efficiency. On the other hand, in the case of dCdapTP with a C3-linker, it is well incorporated against the complementary position of oxodG template DNA (8.00 × 10^6^ %min^−1^M^−1^) (Table 1, entry 6). The higher *V*_max_ value compared to a C2-linker suggests that an effective elongation reaction is occurring (*V*_max_ values: 0.85 %min^−1^ and 2.86 %min^−1^ for the C2-linker and C3-linker, respectively) (Table 1, entries 1 vs. 6). This is expected to result in the formation of an artificial base pair between dCdap with a C3-linker and oxodG. For dG template DNA, almost no differences in parameters are shown at dCdapTP with a C2-linker or C3-linker, which supports the results of the interaction with *anti*-oriented dG with no influence of the linker (for the C2-linker vs. C3-linker, *V*_max_: 1.95 vs. 2.20 %min^−1^, *K*_m_: 0.27 vs. 0.29 µM, respectively) (Table 1, entries 2 vs. 7). Moreover, it is then taken up against T, dC, and dA template DNA (0.64 × 10^6^, 0.50 × 10^6^ and 0.28 × 10^6^ %min^−1^M^−1^, respectively), in that order, but with much lower efficiency. 

## 3. Materials and Methods

### 3.1. General Methods of the Synthesis of Desired Compounds

All starting materials, reagents, and solvents are purchased from Sigma-Aldrich, Inc. (St. Louis, MO, USA) (7M NH_3_ in MeOH (499145). Tributyl ammonium pyrophosphate (P8533)) is from Tokyo Chemical Industry Co., Ltd. (Tokyo, Japan) (3-(Fmoc-amino)-1-propanol (F1036), DIAD (A1246), Et_3_N (T0424), 2,4,6-Triisopropylbenzenesulfonyl chloride (T0459), 1-propanol (P0491), DIPEA (D6179), triethylamine trihydrofluoride (T2022), 2-chloro-4-*H*-1,3,2-benzodioxaphosphorin-4-one (C1210), and tributyl amine (T0357)) are from Nacalai tesque, Inc. (Kyoto, Japan) (PPh_3_ (35312-82), DMAP (12922-86)), ^1^H-NMR (500 MHz), ^13^C-NMR (125 MHz), and ^31^P-NMR (202 MHz) spectra were recorded by Bruker Ascend-500 spectrometers (Billerica, MA, USA), which are shown in the Supplementary Materials. The high-resolution mass spectra were recorded by a Bruker microTOF II. The MALDI-TOF-MS spectra were recorded by a Bruker Autoflex III. The concentration of DNAs was measured by NanoDrop^TM^ One (Thermo Fisher Scientific, Waltham, MA, USA). The gel was visualized by a FUJIFILM Luminoimage analyzer LAS-4000 (Tokyo, Japan).

### 3.2. Synthesis of the Phenoxazine Unit with a C3-Linker (***8***) 

To a solution of compound **6** (1.88 g, 6.03 mmol) in CH_2_Cl_2_ (60 mL), 3-(Fmoc-amino)-1-propanol (2.68 g, 9.00 mmol) and PPh_3_ (2.72 g, 10.4 mmol) were added, and the mixture was stirred at 0 °C under Ar atmosphere. After the addition of DIAD (4.96 mL, 10.1 mmol), the reaction mixture was stirred for 4 h at room temperature. The solvent was removed under reduced pressure, and the residue was purified by column chromatography (NH-silica-gel, CHCl_3_) to obtain compound **7** (2.68 g, 4.52 mmol, 75%) as a yellow solid. This solid was dissolved in 7M NH_3_ in MeOH (200 mL) and DMSO (40 mL), and the reaction mixture was stirred for 13 h at room temperature. The solvent was removed under reduced pressure, and the residue was purified by column chromatography (NH-silica-gel, CH_2_Cl_2_/MeOH = 100/0 to 80/20) to obtain compound **8** (1.21 g, mmol, 93%) as a yellow powder. ^1^H-NMR (500 MHz, CDCl_3_) δ 7.33 (1H, s), 6.76 (1H, t, *J* = 8.2Hz), 6.59 (1H, d, *J* = 7.9Hz), 6.40 (1H, d, *J* = 7.9Hz), 4.03 (2H, s), 3.14 (3H, s), 2.92 (2H, s), 1.14–1.22 (1H, m), 1.09 (1H, d, *J* = 7.0Hz); ^13^C-NMR (126 MHz, DMSO-*d*_6_) δ 153.8, 147.6, 143.0, 127.1, 126.4, 123.1, 121.6, 108.5, 108.0, 67.3, 36.4, 28.9, 24.9; ESI-HRMS (*m*/*z*) calcd. for C_14_H_17_N_4_O_3_ [M + H]^+^: 289.1295, found: 289.1314.

### 3.3. Synthesis of Compound ***11***

To a solution of compound **9** (835 mg, 1.77 mmol) and Et_3_N (1.0 mL, 7.21 mmol) in CH_2_Cl_2_ (8.0 mL), 2,4,6-Triisopropylbenzenesulfonyl chloride (1.1 g, 3.55 mmol) and DMAP (65.0 mg, 0.53 mmol) were added. After stirring for 8 h at room temperature, the reaction mixture was diluted with CHCl_3_ and washed with water and a saturated NaCl solution. The organic layer was dried over Na_2_SO_4_ and removed under reduced pressure. The residue was purified by flush column chromatography (Hexane/EtOAc = 10/1) to obtain compound **11** (892 mg, 1.20 mmol, 68%) as a colorless foam. ^1^H-NMR (500 MHz, CDCl_3_) δ 7.97 (1H, s), 7.19 (2H, s), 6.12 (1H, t, *J* = 6.3 Hz), 4.34–4.31 (1H, m), 4.33–4.29 (2H, m), 3.96 (1H, dt, *J* = 3.4 Hz), 3.89 (1H, dd, *J* = 11.2, 2.4 Hz), 3.74 (1H, dd, *J* = 11.7, 2.4 Hz), 2.92–2.87 (1H, m), 2.51–2.46 (1H, m), 2.03 (3H, s), 1.98–1.93 (1H, m), 1.29 (6H, d, *J* = 6.8 Hz), 1.23 (12H, t, *J* = 7.1 Hz), 0.90–0.87 (18H, m), 0.09–0.04 (12H, m); ESI-HRMS (*m*/*z*) calcd. for C_37_H_65_N_2_O_7_SSi_2_ [M + H]^+^: 737.4046, found: 737.4085. 

### 3.4. Synthesis of Compound ***12***

To a solution of compound **10** (500 mg, 1.10 mmol) and Et_3_N (0.6 mL, 4.33 mmol) in CH_2_Cl_2_ (5.0 mL), 2,4,6-Triisopropylbenzenesulfonyl chloride (663.0 mg, 2.19 mmol) and DMAP (41.0 mg, 0.33 mmol) were added. After stirring for 16 h at room temperature, the reaction mixture was diluted with CHCl_3_ and washed with water and a saturated NaCl solution. The organic layer was dried over Na_2_SO_4_ and removed under reduced pressure. The residue was purified by column chromatography (Hexane/EtOAc = 10/1) to obtain compound **12** (342 mg, 0.47 mmol, 43%) as a colorless foam. ^1^H-NMR (500 MHz, CDCl_3_) δ 8.45 (1H, d, *J* = 7.3 Hz), 7.20 (2H, s), 6.07–6.09 (1H, m), 6.01 (1H, d, *J* = 7.3 Hz), 4.33 (1H, q, *J* = 5.9 Hz), 4.23–4.28 (2H, m), 3.94 (2H, dd, *J* = 11.7, 2.0 Hz), 3.76 (1H, d, *J* = 9.8 Hz), 2.88–2.93 (1H, m), 2.46–2.51 (1H, m), 2.12 (1H, qd, *J* = 6.5, 4.6 Hz), 1.31 (6H, d, *J* = 6.8 Hz), 1.26 (12H, t, *J* = 6.3 Hz), 0.91 (9H, s), 0.86 (9H, s), 0.04–0.10 (12H, m); ESI-HRMS (*m*/*z*) calcd. for C_36_H_62_N_2_O_7_SSi_2_Na [M + Na]^+^: 745.3708, found: 745.3620. 

### 3.5. Synthesis of the TBS-Protected 5-Methyl-Cdap C2-Linker (***13***) 

To a solution of compound **5** (570 mg, 2.08 mmol) in 1-propanol (30 mL) was added compound **11** (1.4 g, 1.87 mmol) and DIPEA (0.3 mL, 1.72 mmol). After stirred for 24 h, the solvent was removed under reduced pressures. The residue was purified by column chromatography (Hexane/EtOAc = 10/1, and then CH_2_Cl_2_/MeOH = 100/1 to 20/1) to obtain compound **13** (609 mg, 1.38 mmol, 74%) as a pale yellow foam. ^1^H-NMR (500 MHz, DMSO-*d*_6_) δ 9.68 (1H, s), 7.51 (1H, s), 7.37 (1H, s), 6.75 (1H, t, *J* = 8.3 Hz), 6.59 (1H, d, *J* = 8.3 Hz), 6.40 (1H, d, *J* = 8.3 Hz), 6.16 (1H, t, *J* = 6.8 Hz), 4.31–4.33 (1H, m), 4.00 (2H, t, *J* = 4.9 Hz), 3.66–3.79 (5H, m), 3.18 (3H, s), 1.99–2.09 (2H, m), 1.89 (3H, s), 0.85 (18H, d, *J* = 6.8 Hz), 0.05 (12H, d, *J* = 7.8 Hz); ^13^C-NMR (126 MHz, DMSO-*d*_6_) δ 162.8, 154.8, 137.3, 126.1, 123.1, 107.9, 107.2, 101.9, 86.9, 84.7, 72.5, 67.1, 62.9, 36.7, 25.8, 18.1, 17.9, 13.5, −4.6, −4.8, −5.3, −5.3; ESI-HRMS (*m*/*z*) calcd. for C_35_H_55_N_6_O_7_Si_2_ [M + H]^+^: 727.3665, found: 727.3647.

### 3.6. Synthesis of the TBS-Protected 5-Methyl-Cdap C3-Linker (***14***) 

To a solution of compound **8** (589 mg, 2.04 mmol) in 1-propanol (50 mL), compound **11** (2.4 g, 3.20 mmol) and DIPEA (0.6 mL, 3.44 mmol) were added. After stirring for 24 h, the solvent was removed under reduced pressure. The residue was purified by column chromatography (Hexane/EtOAc = 10/1, and then CH_2_Cl_2_/MeOH = 50/1 to 10/1) to obtain compound **14** (777 mg, 2.37 mmol, 51%) as a pale yellow foam. ^1^H-NMR (500 MHz, DMSO-*d*_6_) δ 9.79 (1H, s), 7.40 (1H, s), 7.32 (1H, s), 7.22 (1H, s), 6.75 (1H, t, *J* = 8.2 Hz), 6.58 (1H, d, *J* = 8.2 Hz), 6.38 (1H, d, *J* = 7.9 Hz), 6.18 (1H, t, *J* = 6.9 Hz), 4.32–4.34 (1H, m), 3.99 (2H, t, *J* = 5.8 Hz), 3.67–3.78 (3H, m), 3.45–3.52 (2H, m), 3.16 (3H, s), 1.99–2.07 (4H, m), 1.85 (3H, s), 0.86 (18H, s), 0.06 (12H, d, *J* = 4.3 Hz); ^13^C-NMR (126 MHz, DMSO-*d*_6_) δ 162.9, 155.1, 154.1, 153.6, 147.4, 147.0, 142.8, 141.9, 136.7, 126.3, 123.2, 121.5, 107.8, 107.7, 102.1, 86.8, 84.5, 72.5, 66.8, 63.0, 37.8, 36.6, 33.5, 28.2, 27.9, 25.9, 25.9, 25.0, 24.0, 18.2, 17.9, 13.4, −4.6, −4.7, −5.3, −5.3; ESI-HRMS (*m*/*z*) calcd. for C_36_H_56_N_6_O_7_Si_2_Na [M + Na]^+^: 763.3641, found: 763.3677.

### 3.7. Synthesis of the TBS-Protected Cdap C2-Linker (***15***) 

To a solution of compound **5** (570 mg, 2.08 mmol) in 1-propanol (30 mL), compound **12** (2.3 g, 3.13 mmol) and DIPEA (0.5 mL, 3.15 mmol) were added. After stirring for 24 h, the solvent was removed under reduced pressure. The residue was purified by column chromatography (Hexane/EtOAc = 10/1, and then CH_2_Cl_2_/MeOH = 100/1 to 20/1) to obtain compound **15** (1.0 g, 2.09 mmol, 70%) as a pale yellow foam. ^1^H-NMR (500 MHz, DMSO-*d*_6_) δ 9.51 (1H, s), 8.06 (1H, s), 7.69 (1H, d, *J* = 7.3 Hz), 7.49 (1H, s), 6.76 (1H, t, *J* = 8.2 Hz), 6.61 (1H, d, *J* = 8.5 Hz), 6.41 (1H, d, *J* = 8.2 Hz), 6.13 (1H, t, *J* = 6.4 Hz), 5.73 (1H, d, *J* = 7.6 Hz), 4.32–4.35 (1H, m), 3.99 (2H, t, *J* = 4.9 Hz), 3.67–3.79 (5H, m), 3.18 (3H, s), 2.13 (1H, qd, *J* = 6.5, 4.3 Hz), 2.00–2.05 (1H, m), 0.86 (18H, d, *J* = 3.7 Hz), 0.06 (12H, s); ^13^C-NMR (126 MHz, DMSO-*d*_6_) δ 163.3, 154.9, 140.0, 123.1, 108.1, 94.6, 86.7, 84.7, 71.5, 67.4, 62.5, 25.8, 18.1, 17.9, −4.6, −4.8, −5.4, −5.5; ESI-HRMS (*m*/*z*) calcd. for C_34_H_52_N_6_O_7_Si_2_Na [M + Na]^+^: 735.3328, found: 735.3314.

### 3.8. Synthesis of the TBS-Protected Cdap C3-Linker (***16***) 

To a solution of compound **8** (589 mg, 2.04 mmol) in 1-propanol (50 mL), compound **12** (2.3 g, 3.13 mmol) and DIPEA (0.6 mL, 3.44 mmol) were added. After stirring for 24 h, the solvent was removed under reduced pressure. The residue was purified by column chromatography (Hexane/EtOAc = 10/1, and then CH_2_Cl_2_/MeOH = 50/1 to 10/1) to obtain compound **16** (840 mg, 1.16 mmol, 37%) as a pale yellow foam. ^1^H-NMR (500 MHz, DMSO-*d*_6_) δ 7.75 (1H, s), 7.63 (d, *J* = 7.6 Hz, 1H), 6.94 (s, 6H), 6.76 (t, *J* = 8.2 Hz, 1H), 6.60 (d, *J* = 8.2 Hz, 1H), 6.39 (d, *J* = 8.2 Hz, 1H), 6.14 (t, *J* = 6.4 Hz, 1H), 5.72 (d, *J* = 7.6 Hz, 1H), 4.54–4.59 (m, 6H), 4.32–4.34 (m, 1H), 3.99 (t, *J* = 6.0 Hz, 2H), 3.67–3.78 (m, 4H), 3.43 (q, *J* = 6.1 Hz, 1H), 3.16 (s, 3H), 2.76–2.81 (m, 3H), 2.09–2.13 (m, 1H), 1.93–2.03 (m, 3H), 1.15–1.22 (m, 20H), 1.09 (d, *J* = 7.0 Hz, 36H), 0.86 (d, *J* = 2.4 Hz, 18H), 0.08–0.04 (12H); ^13^C-NMR (126 MHz, DMSO-*d*_6_) δ 163.3, 162.2, 154.9, 147.4, 147.2, 146.8, 141.8, 139.2, 121.3, 107.6, 94.6, 86.4, 84.6, 84.5, 71.4, 66.6, 62.3, 37.2, 36.6, 33.3, 28.0, 25.7, 24.8, 23.8, 21.9, 17.9, 17.7, −4.8, −5.0, −5.6, −5.6; ESI-HRMS (*m*/*z*) calcd. for C_35_H_55_N_6_O_7_Si_2_ [M + H]^+^: 727.3665, found: 727.3628.

### 3.9. General Procedure of the Deprotection Reaction in TBS Groups 

To a solution corresponding to a TBS-protected compound (0.4 mmol) in pyridine (5.0 mL), triethylamine trihydrofluoride (0.32 mL, 1.96 mmol) was added. After stirring for 22 h at room temperature, the solution was removed under reduced pressure. The residue was purified by column chromatography (Hexane/EtOAc = 10/1, and then CHCl_3_/MeOH = 50/1 to 10/1) to obtain the corresponding 3′-,5′-diol compound.

^5Me^Cdap C2-linker (**1**) (91%) as a pale yellow foam. ^1^H-NMR (500 MHz, DMSO-*d*_6_) δ 9.69 (1H, s), 7.62 (1H, s), 7.48 (1H, s), 6.75 (1H, t, *J* = 8.3 Hz), 6.59 (1H, d, *J* = 8.3 Hz), 6.40 (1H, d, *J* = 7.8 Hz), 6.15 (1H, t, *J* = 6.8 Hz), 5.15 (1H, d, *J* = 4.4 Hz), 4.96 (1H, t, *J* = 5.1 Hz), 4.17–4.21 (1H, m), 4.00 (2H, t, *J* = 4.6 Hz), 3.73 (3H, q, *J* = 3.6 Hz), 3.50–3.59 (2H, m), 3.18 (3H, s), 2.05 (1H, qd, *J* = 6.5, 3.5 Hz), 1.91–1.97 (1H, m), 1.89 (3H, s); ^13^C-NMR (126 MHz, DMSO-*d*_6_) δ 163.0, 155.1, 137.9, 123.1, 107.9, 107.2, 101.8, 87.3, 84.7, 70.5, 67.1, 61.5, 36.7, 13.5; ESI-HRMS (*m*/*z*) calcd. for C_23_H_26_N_6_O_7_Na [M + Na]^+^: 521.1755, found: 521.1726.

^5Me^Cdap C3-linker (**2**) (79%) as a pale yellow foam. ^1^H-NMR (500 MHz, DMSO-*d*_6_) δ 9.76 (1H, s), 7.56 (1H, s), 7.39 (1H, s), 7.13 (1H, s), 6.77 (1H, t, *J* = 8.3 Hz), 6.60 (1H, d, *J* = 8.3 Hz), 6.39 (1H, d, *J* = 8.3 Hz), 6.16 (1H, t, *J* = 6.8 Hz), 5.14 (1H, d, *J* = 4.4 Hz), 4.95 (1H, t, *J* = 5.1 Hz), 4.17–4.21 (1H, m), 4.00 (2H, t, *J* = 6.1 Hz), 3.73 (1H, q, *J* = 3.4 Hz), 3.41–3.59 (4H, m), 3.16 (3H, s), 1.93–2.06 (2H, m), 1.84 (3H, s), 1.14–1.26 (1H, m), 1.03–1.10 (1H, m); ^13^C-NMR (126 MHz, DMSO-*d*_6_) δ 162.8, 155.2, 142.6, 137.3, 126.1, 123.0, 107.7, 107.6, 101.7, 87.1, 84.6, 70.4, 66.6, 61.4, 56.0, 45.7, 37.6, 36.4, 27.8, 18.6, 13.2; ESI-HRMS (*m*/*z*) calcd. for C_24_H_28_N_6_O_7_Na [M + Na]^+^: 535.1912, found: 535.1899.

Cdap C2-linker (**3**) (94%) as a pale yellow foam. ^1^H-NMR (500 MHz, DMSO-*d*_6_) δ 9.57 (1H, s), 8.04 (1H, s), 7.78 (1H, d, *J* = 7.6 Hz), 7.48 (1H, s), 6.77 (1H, t, *J* = 8.2 Hz), 6.62 (1H, d, *J* = 7.9 Hz), 6.42 (1H, d, *J* = 8.2 Hz), 6.15 (1H, t, *J* = 6.7 Hz), 5.77 (1H, d, *J* = 7.3 Hz), 5.17 (1H, d, *J* = 4.3 Hz), 4.94 (1H, t, *J* = 5.3 Hz), 4.18 (1H, dt, *J* = 9.4, 3.4 Hz), 3.99 (2H, t, *J* = 5.0 Hz), 3.75 (1H, q, *J* = 3.6 Hz), 3.66–3.70 (2H, m), 3.50–3.57 (2H, m), 3.18 (3H, s), 2.09 (1H, qd, *J* = 13.2, 3.1 Hz), 1.89–1.95 (1H, m); ^13^C-NMR (126 MHz, DMSO-*d*_6_) δ 163.3, 155.1, 140.5, 126.0, 123.2, 108.1, 94.8, 87.4, 85.1, 70.6, 67.4, 61.6, 36.7; ESI-HRMS (*m*/*z*) calcd. for C_22_H_24_N_6_O_7_Na [M + Na]^+^: 507.1599, found: 507.1580.

Cdap C3-linker (**4**) (85%) as a pale yellow foam. ^1^H-NMR (500 MHz, DMSO-*d*_6_) δ 9.16 (s, 1H), 7.77 (s, 1H), 7.72 (d, *J* = 7.3 Hz, 1H), 6.94 (s, 6H), 6.77 (t, *J* = 8.2 Hz, 1H), 6.60 (d, *J* = 8.2 Hz, 1H), 6.39 (d, *J* = 7.9 Hz, 1H), 6.14 (t, *J* = 6.6 Hz, 1H), 5.75 (d, *J* = 7.6 Hz, 1H), 5.17 (d, *J* = 3.4 Hz, 1H), 4.94 (s, 1H), 4.53–4.58 (m, 6H), 4.18 (s, 1H), 3.99 (t, *J* = 5.6 Hz, 2H), 3.74 (d, *J* = 2.7 Hz, 1H), 3.51–3.56 (m, 1H), 3.43 (d, *J* = 5.8 Hz, 1H), 3.16 (s, 3H), 3.08 (q, *J* = 3.4 Hz, 2H), 2.76–2.81 (m, 3H), 2.06–2.10 (m, 1H), 1.88–1.97 (m, 3H), 1.14–1.18 (m, 21H), 1.09 (d, *J* = 6.7 Hz, 38H); ^13^C-NMR (126 MHz, DMSO-*d*_6_) δ 155.0, 147.2, 146.8, 141.8, 139.8, 121.5, 107.6, 94.7, 87.2, 84.8, 70.4, 69.8, 66.5, 61.4, 45.7, 37.1, 36.4, 33.3, 28.0, 27.7, 24.8, 23.9, 8.6; ESI-HRMS (*m*/*z*) calcd. for C_23_H_26_N_6_O_7_Na [M + Na]^+^: 521.1755, found: 521.1751

### 3.10. General Procedure of the Synthesis of the 5′-Triphosphate Compound 

The solution of 2-chloro-4-*H*-1,3,2-benzodioxaphosphorin-4-one (0.13 mmol) in DMF (0.17 mL) and tributyl amine (0.25 mL) was added dropwise to the solution of tributyl ammonium pyrophosphate (0.13 mmol) in DMF (0.17 mL) and stirred for 30 min under an argon atmosphere at room temperature. This reaction mixture was added to the solution of the corresponding 3′-,5′-diol compounds (**1**, **2**, **3**, or **4**) (0.05 mmol) in DMF (0.35 mL). After stirring for 2 h, the oxidation reagent (2.0 mL, 1.0% I_2_ in pyridine/H_2_O = 98/2) was added to the reaction mixture and stirred for 30 min, and then the reaction was quenched by 5.0% NaHSO_3_ solution (1.5 mL). After ethanol precipitation, the resulting residue was purified by reverse-phase HPLC to give corresponding 5′-triphosphate compounds as a white material after lyophilization. (HPLC conditions: A: 20 mM TEAA, B: MeCN; flow rate: 1.0 mL/min; COSMOSIL 5C18-AR-II (4.6 ID × 250 mm); FL detector: ex: 365 nm, em: 452 nm; 35 °C.)

^5Me^dCdapTP C2-linker (**17**) (2%). ^1^H-NMR (500 MHz, D_2_O) δ 7.61 (1H, s), 7.25 (1H, s), 6.88 (1H, t, *J* = 8.4 Hz), 6.75 (1H, d, *J* = 7.6 Hz), 6.47 (1H, d, *J* = 9.2 Hz), 6.21 (1H, t, *J* = 6.6 Hz), 4.66–4.63 (1H, m), 4.45–4.40 (2H, m), 4.28–4.18 (2H, m), 4.16–4.11 (1H, m), 3.98–3.93 (1H, m), 3.80–3.75 (1H, m), 3.35 (3H, s), 2.36–2.31 (1H, m), 2.25–2.20 (1H, m), 1.85 (3H, s); ^31^P-NMR (202 MHz, D_2_O) δ −5.99 (d, *J* = 19.9 Hz), −11.28 (d, *J* = 19.9 Hz), −21.93 (t, *J* = 19.9 Hz), ESI-HRMS (*m*/*z*) calcd. for C_23_H_28_N_6_O_16_P_3_ [M − H]^−^: 737.0769, found: 737.0737. 

^5Me^dCdapTP C3-linker (**18**) (12%). ^1^H-NMR (500 MHz, D_2_O) δ 7.56 (1H, s), 7.16 (1H, s), 6.80 (1H, t, *J* = 8.4 Hz), 6.55 (1H, d, *J* = 8.5 Hz), 6.36 (1H, d, *J* = 8.2 Hz), 6.18 (1H, t, *J* = 6.7 Hz), 4.63–4.60 (1H, m), 4.26–4.16 (4H, m), 4.15–4.13 (1H, m), 3.64 (2H, ddd, *J* = 23.9, 13.8, 6.0 Hz), 3.30 (3H, s), 2.35–2.30 (1H, m), 2.25–2.20 (1H, m), 1.93 (3H, s), 1.86 (2H, s); ^31^P-NMR (202 MHz, D_2_O) δ −5.80 (d, *J* = 19.5 Hz), −11.18 (d, *J* = 19.5 Hz), −21.70 (t, *J* = 19.5 Hz), ESI-HRMS (*m*/*z*) calcd. for C_24_H_30_N_6_O_16_P_3_ [M − H]^−^: 751.0926, found: 751.0958. 

dCdapTP C2-linker (**19**) (7%). ^1^H-NMR (500 MHz, D_2_O) δ 7.81 (1H, d, *J* = 7.6 Hz), 7.17 (1H, s), 6.87 (1H, t, *J* = 8.2 Hz), 6.70 (1H, d, *J* = 8.2 Hz), 6.45 (1H, d, *J* = 8.2 Hz), 6.24 (1H, t, *J* = 6.6 Hz), 6.05 (1H, d, *J* = 7.6 Hz), 4.65–4.62 (1H, m), 4.12–4.33 (5H, m), 3.80–3.85 (1H, m), 3.75–3.69 (1H, m), 3.31 (3H, s), 2.39–2.34 (1H, m), 2.27–2.22 (1H, m); ^31^P-NMR (202 MHz, D_2_O) δ −5.88 (d, *J* = 19.9 Hz), −11.05 (d, *J* = 19.9 Hz), −21.79 (t, *J* = 19.9 Hz), ESI-HRMS (*m*/*z*) calcd. for C_22_H_26_N_6_O_16_P_3_ [M − H]^−^: 723.0613, found: 723.0612. 

dCdapTP C3-linker (**20**) (10%). ^1^H-NMR (500 MHz, D_2_O) δ 7.77 (1H, d, *J* = 7.6 Hz), 7.29 (1H, s), 6.87 (1H, t, *J* = 8.4 Hz), 6.65 (1H, d, *J* = 8.2 Hz), 6.45 (1H, d, *J* = 7.9 Hz), 6.21 (1H, t, *J* = 6.6 Hz), 5.97 (1H, d, *J* = 7.3 Hz), 4.64–4.61 (1H, m), 4.27–4.17 (3H, m), 3.58 (2H, t, *J* = 5.8 Hz), 3.36 (3H, s), 2.39–2.34 (1H, m), 2.26–2.18 (1H, m), 2.13–2.10 (2H, m), 1.35–1.30 (2H, m); ^31^P-NMR (202 MHz, D_2_O) δ −6.12 (d, *J* = 19.9 Hz), −11.18 (d, *J* = 19.9 Hz), −22.17 (t, *J* = 19.9 Hz), ESI-HRMS (*m*/*z*) calcd. for C_23_H_28_N_6_O_16_P_3_ [M − H]^−^: 737.0769, found: 737.0813. 

## 4. Conclusions

In this study, we designed and successfully synthesized the oxodG recognition molecules dCdapTP and ^5Me^dCdapTP. Unfortunately, ^5Me^dCdapTP was not incorporated into the primer strand for oxodG template DNA in the single nucleotide elongation reaction using DNA polymerase. On the other hand, dCdapTP derivatives without a methyl group were incorporated into the primer strand of the template strand of oxodG and KF. In particular, the dCTP derivative with a propylene linker was more efficiently incorporated into the primer strand against the oxodG of the template DNA than those with an ethylene linker. Further development of derivatives based on this molecular design would enable the development of novel nucleotide derivatives with enhanced selectivity for recognition against oxodG template DNA. Therefore, we are currently attempting to synthesize such derivatives.

## Figures and Tables

**Table 1:** Steady-state kinetic parameters.

Entry	dNTP	X=	*V*_max_ (%min^−1^)	*K*_m_ (µM)	Efficiency (%min^−1^M^−1^)
1	dCdapTP C2-linker	8-oxodG	0.85	0.29	2.91 × 10^6^
2		dG	1.95	0.27	7.22 × 10^6^
3		dA	0.09	0.13	0.69 × 10^6^
4		dC	0.08	0.07	1.13 × 10^6^
5		T	0.11	0.13	0.88 × 10^6^
6	dCdapTP C3-linker	8-oxodG	2.86	0.36	8.00 × 10^6^
7		dG	2.20	0.28	7.64 × 10^6^
8		dA	0.06	0.23	0.28 × 10^6^
9		dC	0.07	0.15	0.50 × 10^6^
10		T	0.09	0.14	0.64 × 10^6^

Conditions: 1.0 µM 15 mer/25 mer FAM-labeled primer template duplex, 0.01–0.04 unit/μL Klenow Fragment, 20 mM Tris-HCl (pH 7.9), 50 mM NaCl, 10 mM MgCl_2_, 1 mM DTT, dNTPs, incubated at 37 °C for 1–20 min in a reaction volume of 10 μL. Values are given as the mean of three independent experiments. Velocity is normalized for the lowest enzyme concentration used.

## Data Availability

Data are contained within the article and Supplementary Materials.

## Supplementary Materials

The following supporting information can be downloaded at https://www.mdpi.com/article/10.3390/molecules29102270/s1, NMR spectra of new compounds.

## References

[1] Betteridge D.J. (2000). What is oxidative stress?. Metabolism.

[2] Nakabeppu Y., Behmanesh M., Yamaguchi H., Yoshimura D., Sakumi K., Evans M.D., Cooke M.S. (2007). Oxidative Damage to Nucleic Acids.

[3] Seidel C.A., Schulz A., Sauer M.H. (1996). Nucleobase-Specific Quenching of Fluorescent Dyes. 1. Nucleobase One-Electron Redox Potentials and Their Correlation with Static and Dynamic Quenching Efficiencies. J. Phys. Chem..

[4] Steenken S., Jovanovic S.V. (1997). How Easily Oxidizable Is DNA? One-Electron Reduction Potentials of Adenosine and Guanosine Radicals in Aqueous Solution. J. Am. Chem. Soc..

[5] Burrows C.J., Muller J.G. (1998). Oxidative Nucleobase Modifications Leading to Strand Scission. Chem. Rev..

[6] Greenberg M.M. (2009). Radical and Radical Ion Reactivity in Nucleic Acid Chemistry.

[7] Kino K., Kawada T., Hirao-Suzuki M., Morikawa M., Miyazawa H. (2020). Products of oxidative guanine damage form base pairs with guanine. Int. J. Mol. Sci..

[8] Chatterjee N., Walker G.C. (2017). Mechanisms of DNA damage, repair, and mutagenesis. Environ. Mol. Mutagen..

[9] Santivasi W.L., Xia F. (2014). Ionizing radiation-induced DNA damage, response, and repair. Antioxid. Redox Signal..

[10] Ames B.N., Gold L.S. (1991). Endogenous mutagens and the causes of aging and cancer. Mutat. Res..

[11] Steenken S. (1989). Purine bases, nucleosides, and nucleotides: Aqueous solution redox chemistry and transformation reactions of their radical cations and e- and OH adducts. Chem. Rev..

[12] Mingard C., Wu J., McKeague M., Sturla S.J. (2020). Next-generation DNA damage sequencing. Chem. Soc. Rev..

[13] Simoeki A., Rytarowska A., Poplawska A.S., Gackowski D., Rozalski R., Dziaman T., Szaflarska M.C., Olinski R. (2006). Helicobacter pylori infection is associated with oxidatively damaged DNA in human leukocytes and decreased level of urinary 8-oxo-7,8-dihydroguanine. Carcinogenesis.

[14] Hah S.S., Mundt J.M., Kim H.M., Sumbad R.A., Turteltaub K.W., Henderson P.T. (2007). Measurement of 7,8-dihydro-8-oxo-2-deoxyguanosine metabolism in MCF-7 cells at low concentrations using accelerator mass spectrometry. Proc. Natl. Acad. Sci. USA.

[15] Nakae Y., Stoward P.J., Bespalov I.A., Melamede R.J., Wallace S.S. (2005). A new technique for the quantitative assessment of 8-oxoguanine in nuclear DNA as a marker of oxidative stress. Application to dystrophin-deficient DMD skeletal muscles. Histochem. Cell Biol..

[16] Lin K.Y., Matteucci M.D. (1998). A cytosine analogue capable of clamp-like binding to a guanine in helical nucleic acids. J. Am. Chem. Soc..

[17] Bajacan J.E.V., Hong I.S., Penning T.W., Greenberg M.M. (2004). Quantitative detection of 8-oxo-7,8-dihydro-2′-deoxyguanosine using chemical tagging and qPCR. Chem. Res. Toxicol..

[18] Zhang B., Guo L.H., Greenberg M.M. (2012). Quantification of 8-oxodguo lesions in double-stranded DNA using a photoelectrochemical DNA sensor. Anal. Chem..

[19] Schibel A.E.P., An N., Jin Q., Fleming A.M., Burrows C.J., White H.S. (2010). Nanopore detection of 8-oxo-7,8-dihydro-2′-deoxyguanosine in immobilized single-stranded DNA via adduct formation to the DNA damage site. J. Am. Chem. Soc..

[20] Riedl J., Fleming A.M., Burrows C.J. (2016). Sequencing of DNA lesions facilitated by site-specific excision via base excision repair DNA glycosylases yielding ligatable gaps. J. Am. Chem. Soc..

[21] Fromme J.C., Bruner S.D., Yang W., Karplus M., Verdine G.L. (2003). Product-assisted catalysis in base-excision DNA repair. Nat. Struct. Mol. Biol..

[22] Nash H.M., Lu R., Lane W.S., Verdine G.L. (1997). The critical active-site amine of the human 8-oxoguanine DNA glycosylase, hOgg1: Direct identification, ablation and chemical reconstitution. Chem Biol..

[23] Lu R., Nash H.M., Verdine G.L. (1997). A mammalian DNA repair enzyme that excises oxidatively damaged guanines maps to a locus frequently lost in lung cancer. Curr. Biol..

[24] Taniguchi Y., Kawaguchi R., Sasaki S. (2011). Adenosine-1,3-diazaphenoxazine Derivative for Selective Base Pair Formation with 8-Oxo-2′-deoxyguanosine in DNA. J. Am. Chem. Soc..

[25] Taniguchi Y., Kikukawa Y., Sasaki S. (2015). Discrimination between 8-oxo-2′-deoxyguanosine and 2′-deoxyguanosine in DNA by the single nucleotide primer extension reaction with Adap triphosphate. Angew. Chem. Int. Ed..

[26] Kikukawa Y., Kawazoe R., Miyahara R., Sakurada T., Nagata Y., Sasaki S., Taniguchi Y. (2022). Multiple-turnover single nucleotide primer extension reactions to detect of 8-oxo-2′-deoxyguanosine. ChemComm.

[27] Ohkubo A., Yamada K., Ito Y., Yoshimura K., Miyauchi K., Kanamori T., Masaki Y., Seio K., Yuasa H., Sekine M. (2015). Synthesis and triplex-forming properties of oligonucleotides capable of recognizing corresponding DNA duplexes containing four base pairs. Nucleic Acids Res..

[28] Steinert H.S., Schäfer F., Jonker H.R.A., Heckel A., Schwalbe H. (2014). Influence of the absolute configuration of NPE-caged cytosine on DNA single base pair stability. Angew. Chem. Int. Ed..

[29] Caton-Williams J., Hoxhaj R., Fiaz B., Huang Z. (2013). Use of a novel 5′-regioselective phosphitylating reagent for one-pot synthesis of nucleoside 5′-triphosphates from unprotected nucleosides. Curr. Protoc. Nucleic Acid Chem..

