# Supplementary material for: Recognition of 8-Oxo-2′-deoxyguanosine in DNA Using the Triphosphate of 2′-Deoxycytidine Connecting the 1,3-Diazaphenoxazine Unit, dCdapTP
Source: Molecules. 2024 May 11;29(10):2270. doi: 10.3390/molecules29102270 (PMC11123937; doi:10.3390/molecules29102270)

Supporting information (SI)

Recognition of 8-Oxo-2′-Deoxyguanosine in DNA Using the Triphosphate of 2′-Deoxycytidine Connecting the 1,3-Diazaphenoxazine Unit, dCdapTP

Takato Sakurada [1], Yuta Chikada [1,2], Ryo Miyahara [1] and Yosuke Taniguchi [1,2,*]

1 Graduate School of Pharmaceutical Sciences, Kyushu University, 3-1-1 Maidashi, Higashi-ku, Fukuoka 812-8582, Japan; takatorres.31@gmail.com (T.S.); chikada.yuta.840@s.kyushu-u.ac.jp (Y.C.); c51339rm@gmail.com (R.M.)

2 Faculty of Medicine, Dentistry and Pharmaceutical Sciences, Okayama University, 1-1-1 Tsushima-naka, Kita-ku, Okayama 700-8530, Japan

* Correspondence: y-taniguchi@okayama-u.ac.jp; Tel.: +81-86-251-7964

Contents:

1. NMR spectra of new compounds (1–4, 8, 13–16) p2
2. NMR spectra of corresponding triphosphate compounds (17–20) p12

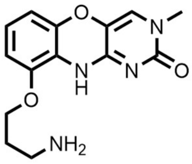
O
N
N
O
H
O
NH2

Compound 8

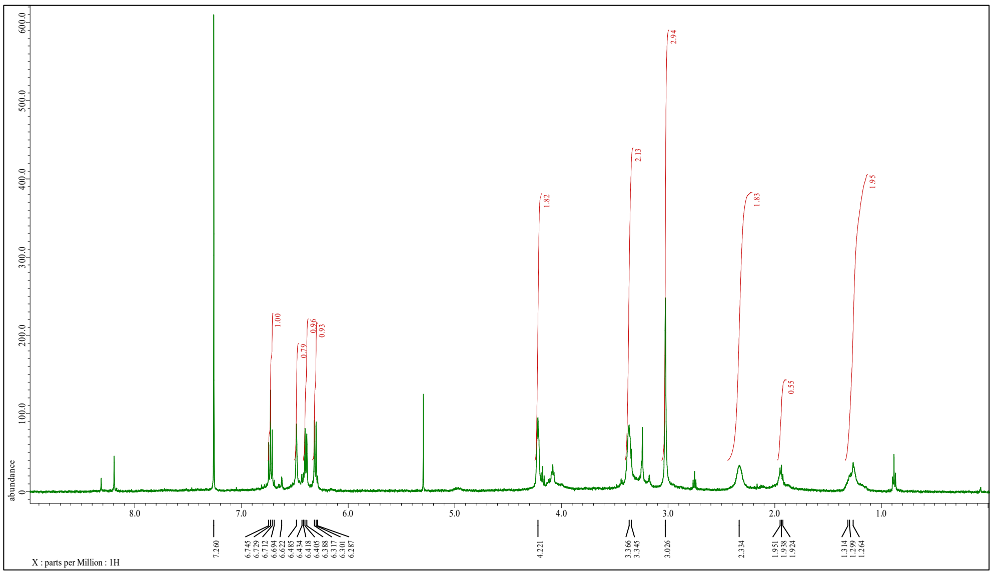
abundance
600.0
500.0
400.0
300.0
200.0
100.0
0
1.00
0.79
0.96
0.93
1.82
2.13
2.94
1.83
0.55
1.95
8.0
7.0
6.0
5.0
4.0
3.0
2.0
1.0
7.260
6.745
6.729
6.712
6.694
6.622
6.485
6.434
6.418
6.405
6.388
6.317
6.301
6.287
4.221
3.366
3.345
3.026
2.334
1.951
1.938
1.924
1.314
1.299
1.264
X : parts per Million : 1H

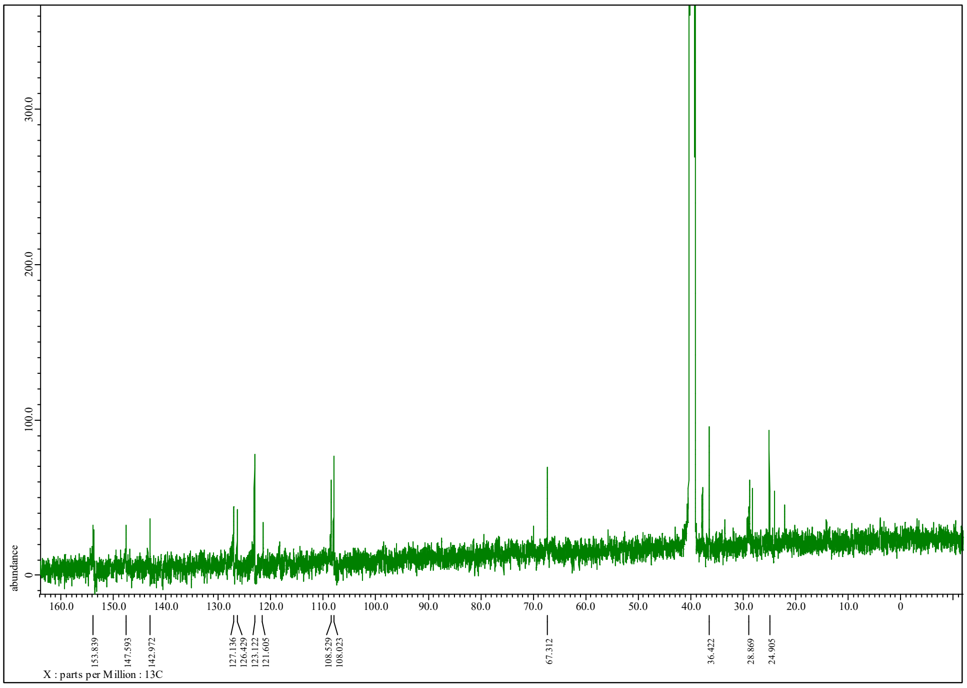
abundance
300.0
200.0
100.0
0
160.0
150.0
140.0
130.0
120.0
110.0
100.0
90.0
80.0
70.0
60.0
50.0
40.0
30.0
20.0
10.0
0
153.839
147.593
142.972
127.136
126.429
123.122
121.605
108.529
108.023
67.312
36.422
28.869
24.905
X : parts per Million : 13C

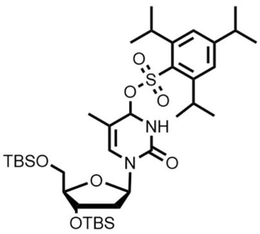

Compound 11

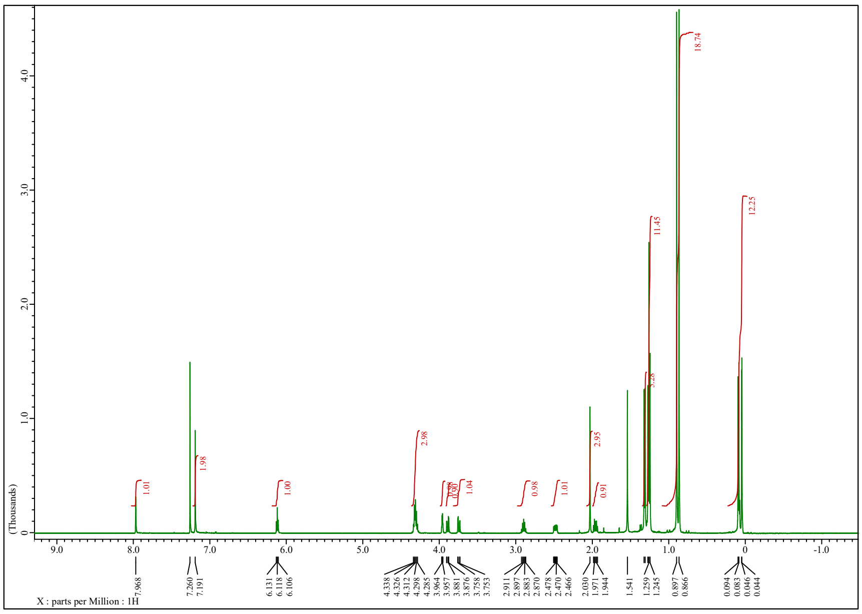

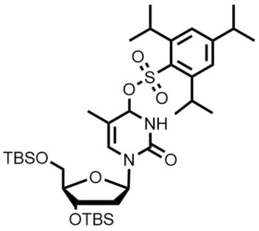

Compound 12

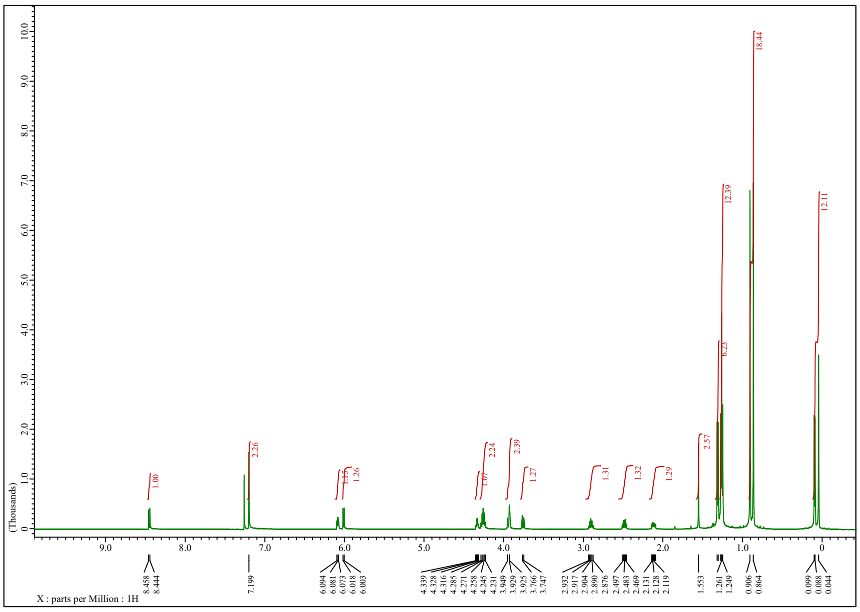

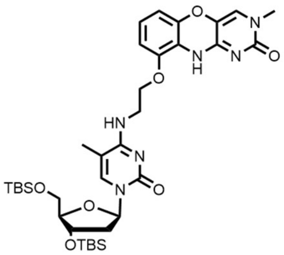

TBSO
O
N
O
N
HN
O
N
H
N
O
OTBS

Compound 13

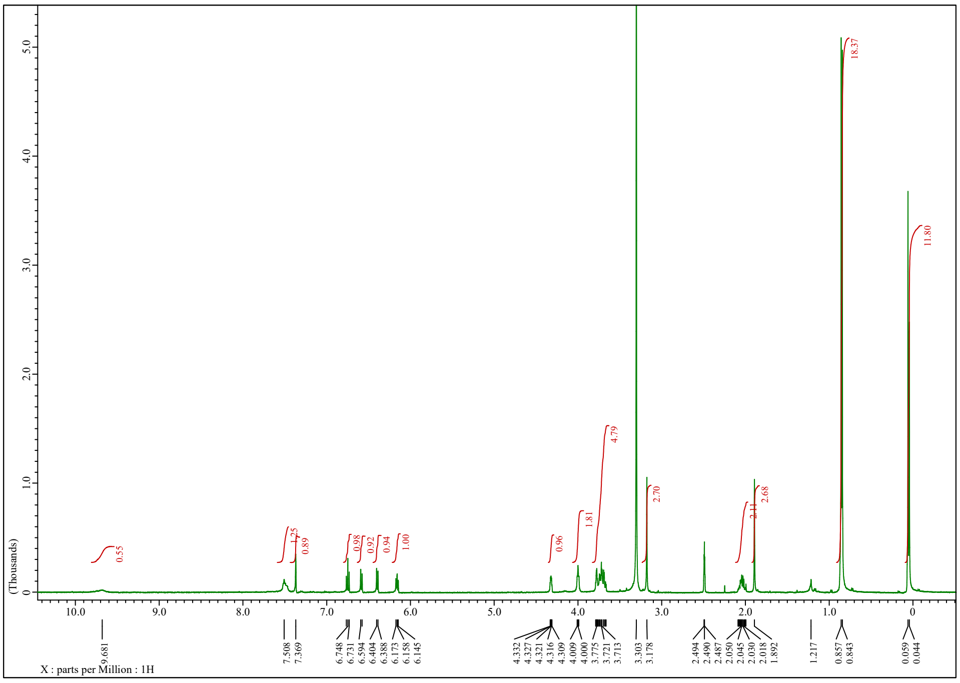

(Thousands)
0.55
1.25
0.89
0.98
0.92
0.94
1.00
0.96
1.81
4.79
2.70
2.11
2.68
18.37
11.80
9.681
7.508
7.369
6.748
6.731
6.594
6.404
6.388
6.173
6.158
6.145
4.332
4.327
4.321
4.316
4.309
4.009
4.000
3.775
3.721
3.713
3.303
3.178
2.494
2.490
2.487
2.050
2.045
2.030
2.018
1.892
1.217
0.857
0.843
0.059
0.044
X : parts per Million : 1H

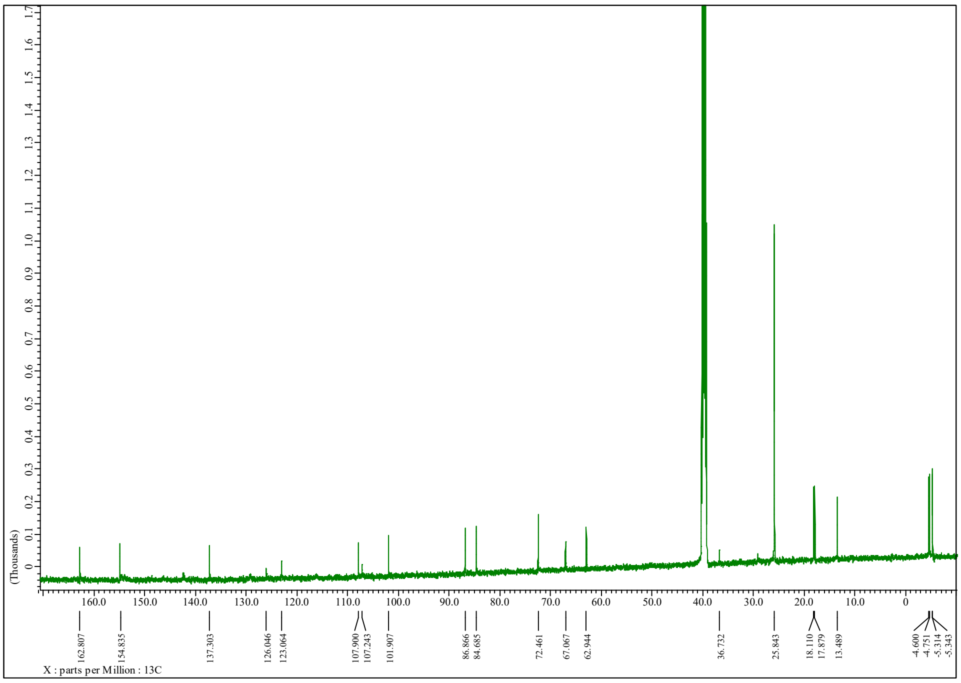

(Thousands)
162.807
154.835
137.303
126.046
123.064
107.900
107.243
101.907
86.866
84.685
72.461
67.067
62.944
36.732
25.843
18.110
17.879
13.489
-4.600
-4.751
-5.314
-5.343
X : parts per Million : 13C

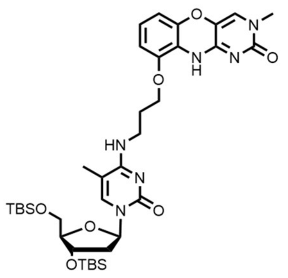
TBSO
OTBS
HN
N
O
N
H

Compound 14

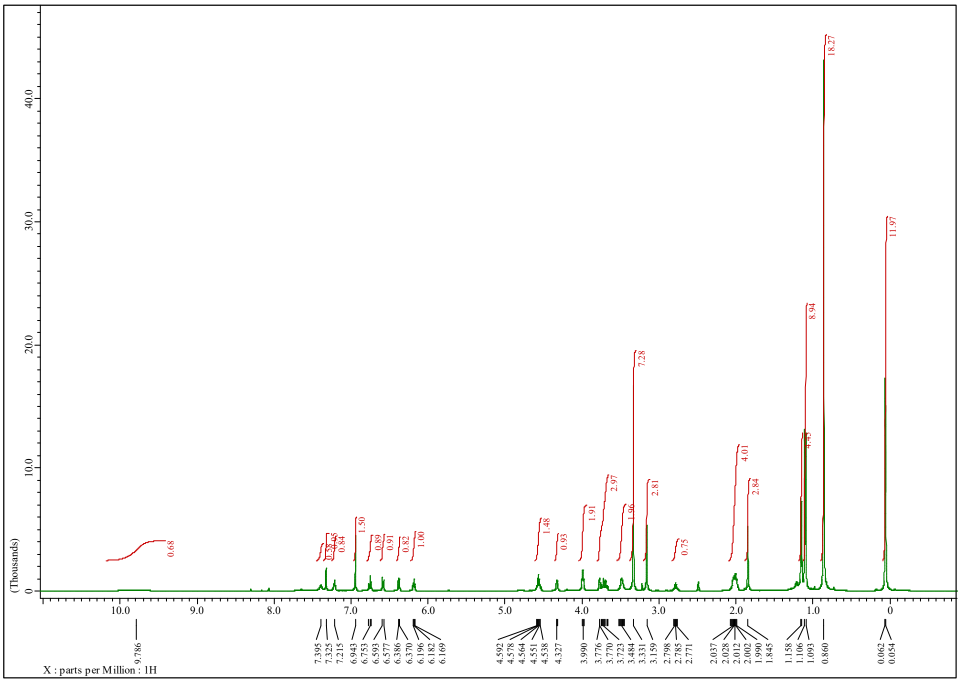
(Thousands)
X : parts per Million : 1H
9.786
7.395
7.325
7.215
6.943
6.753
6.593
6.577
6.386
6.370
6.196
6.182
6.169
4.592
4.578
4.564
4.551
4.538
4.327
3.990
3.776
3.770
3.723
3.484
3.331
3.159
2.798
2.785
2.771
2.037
2.028
2.012
2.002
1.990
1.845
1.158
1.106
1.093
0.860
0.062
0.054

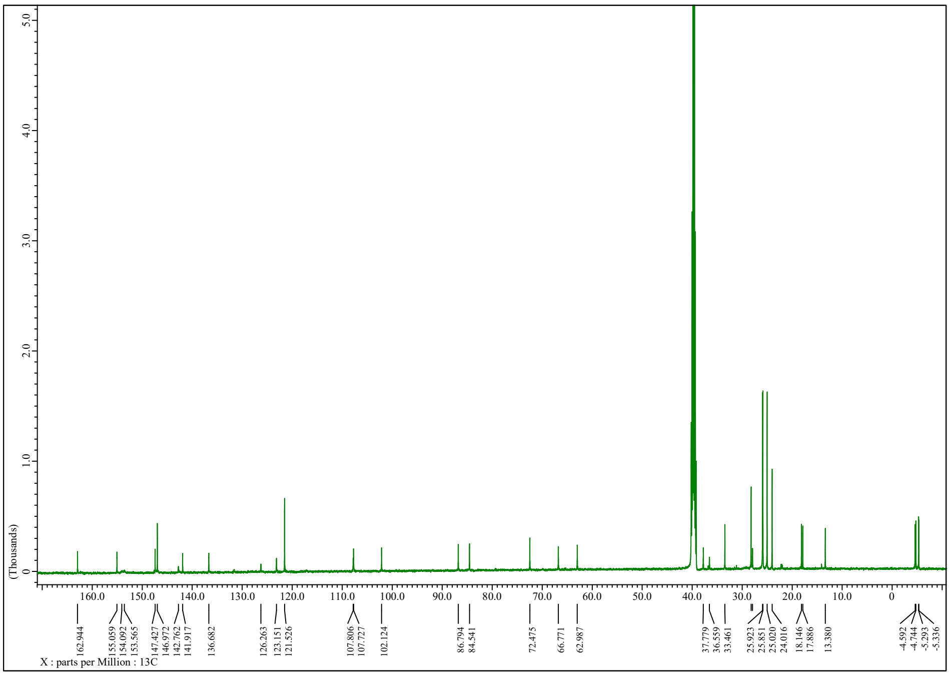
(Thousands)
X : parts per Million : 13C
162.944
155.059
154.092
153.565
147.427
146.972
142.762
141.917
136.682
126.263
123.151
121.526
107.806
107.727
102.124
86.794
84.541
72.475
66.771
62.987
37.779
36.559
33.461
25.923
25.851
25.020
24.016
18.146
17.886
13.380
-4.592
-4.744
-5.293
-5.336

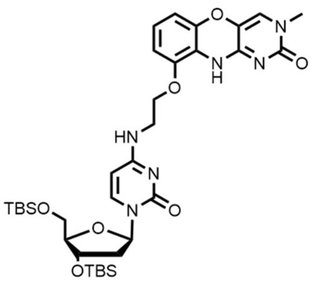
TBSO
OTBS
HN

Compound 15

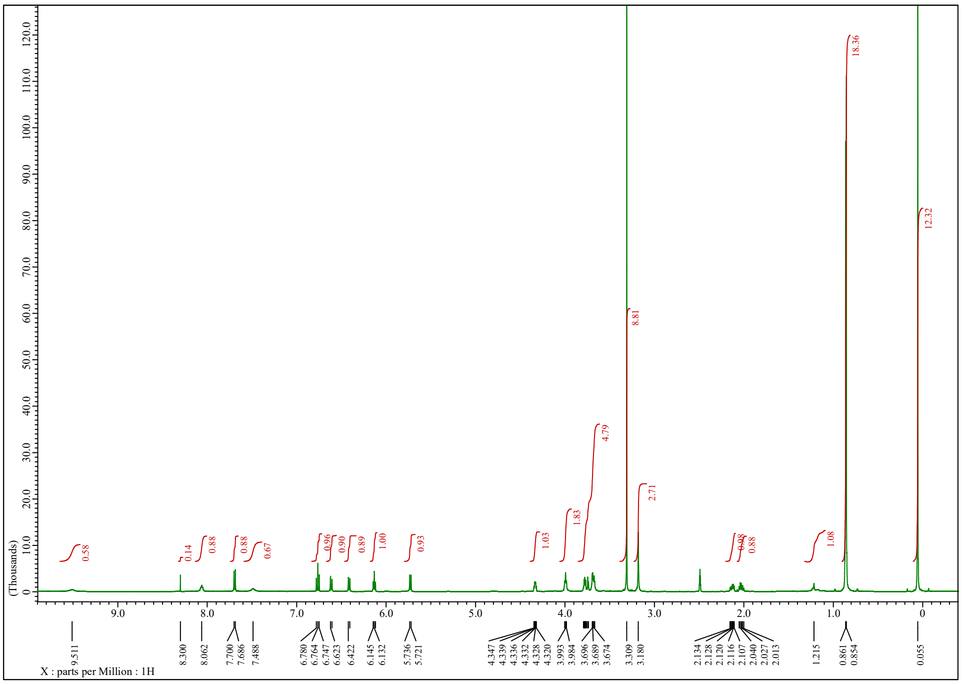
(Thousands)
X : parts per Million : 1H
9.511
8.300
8.062
7.700
7.686
7.488
6.780
6.764
6.747
6.623
6.422
6.145
6.132
5.736
5.721
4.347
4.339
4.336
4.332
4.328
4.320
3.993
3.984
3.696
3.689
3.674
3.309
3.180
2.134
2.128
2.120
2.116
2.107
2.040
2.027
2.013
1.215
0.861
0.854
0.055

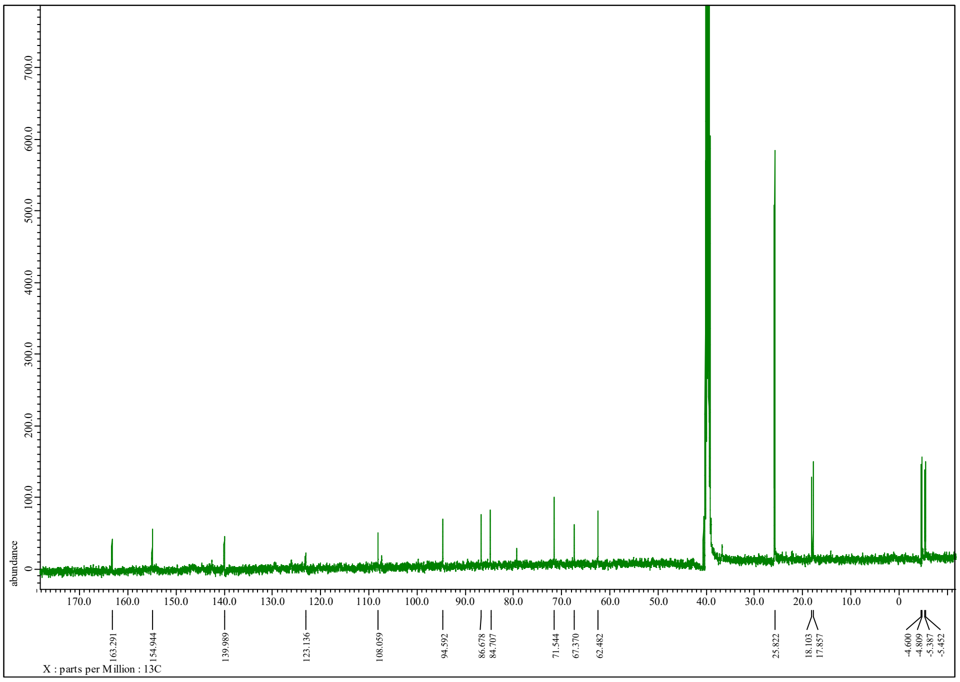
abundance
X : parts per Million : 13C
163.291
154.944
139.989
123.136
108.059
94.592
86.678
84.707
71.544
67.370
62.482
25.822
18.103
17.857
-4.600
-4.809
-5.387
-5.452

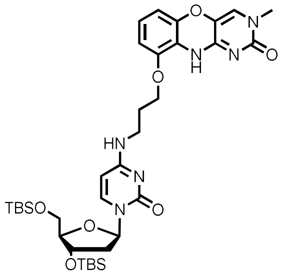

Compound **16**

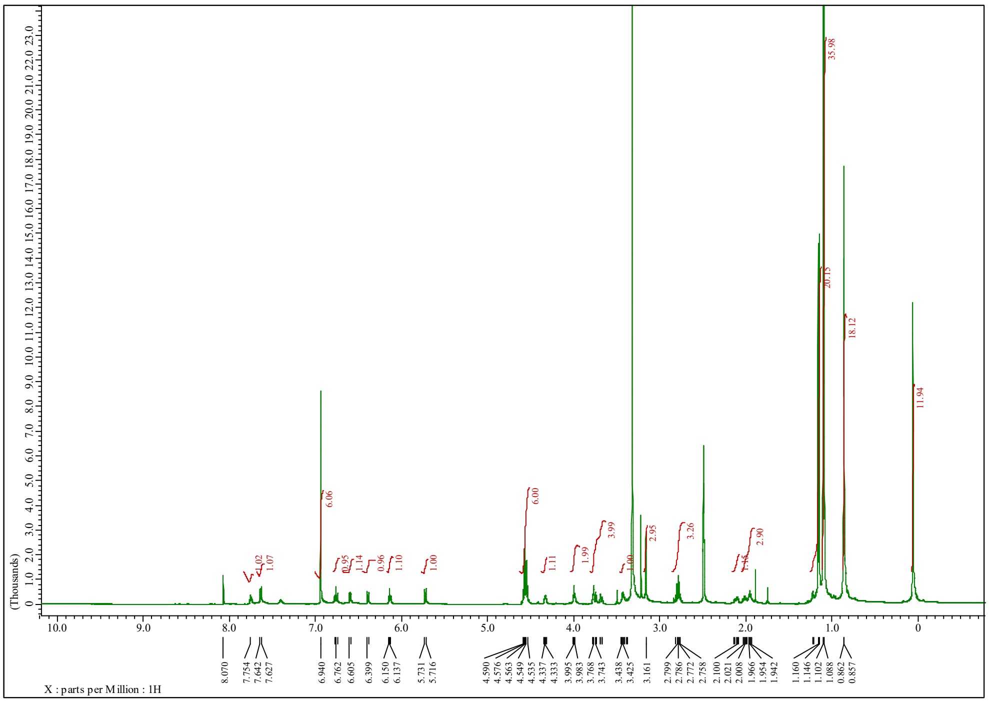

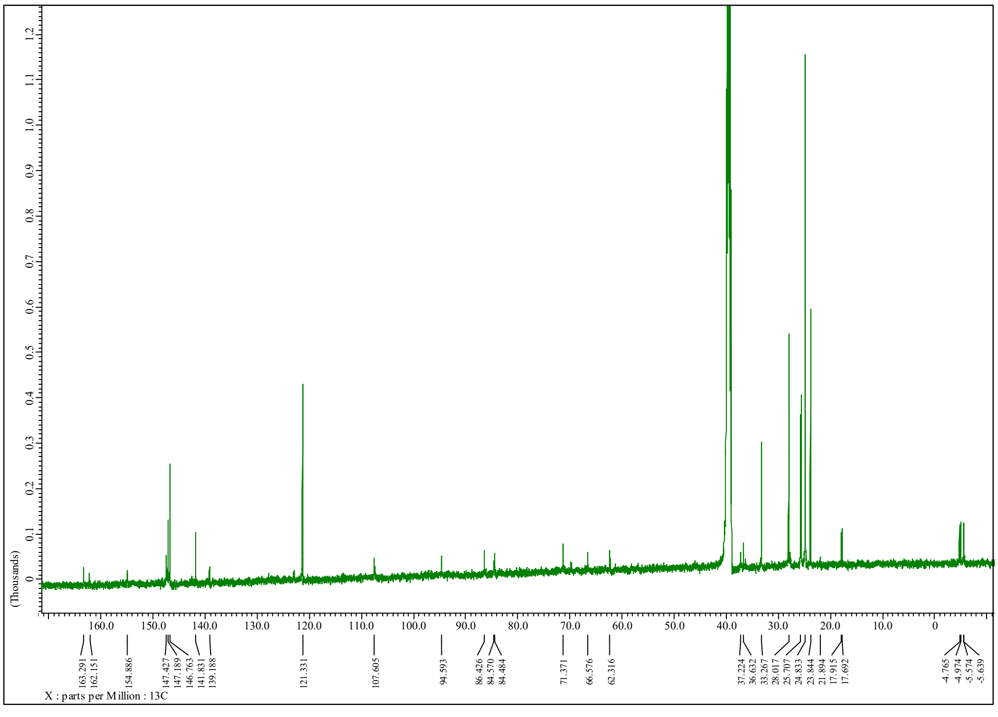

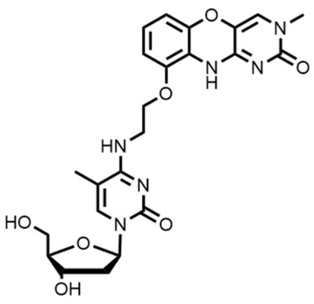

Compound 1

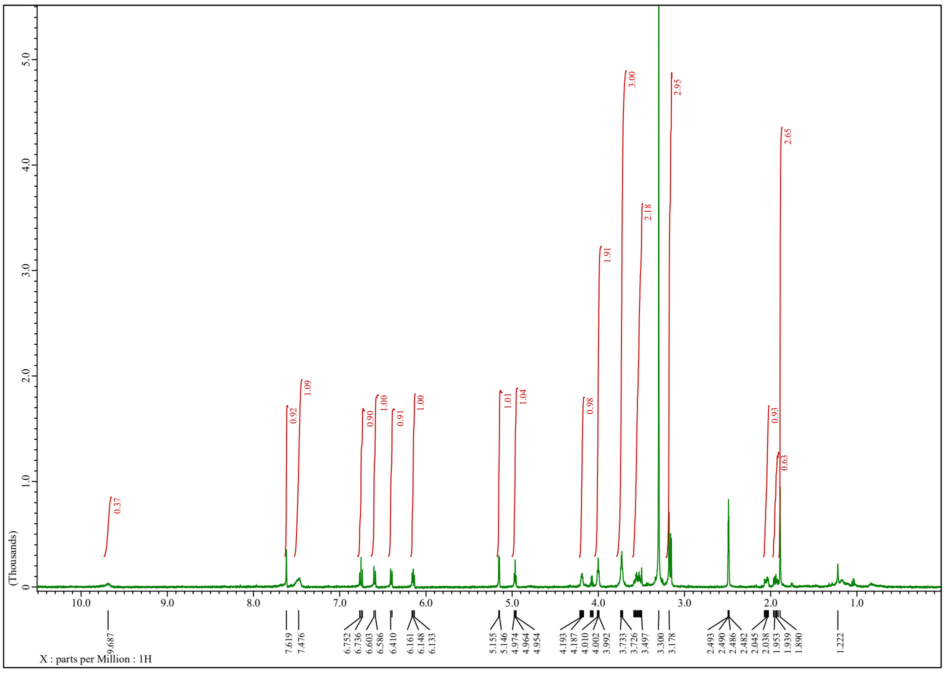

(Thousands)
5.0
4.0
3.0
2.0
1.0
0
10.0
9.0
8.0
7.0
6.0
5.0
4.0
3.0
2.0
1.0
0.37
0.92
1.09
0.90
1.00
0.91
1.00
1.01
1.04
0.98
1.91
3.00
2.18
2.95
0.93
0.63
2.65
9.687
7.619
7.476
6.752
6.736
6.603
6.586
6.410
6.161
6.148
6.133
5.155
5.146
4.974
4.964
4.954
4.193
4.187
4.010
4.002
3.992
3.733
3.726
3.497
3.300
3.178
2.493
2.490
2.486
2.482
2.045
2.038
1.953
1.939
1.890
1.222
X : parts per Million : 1H

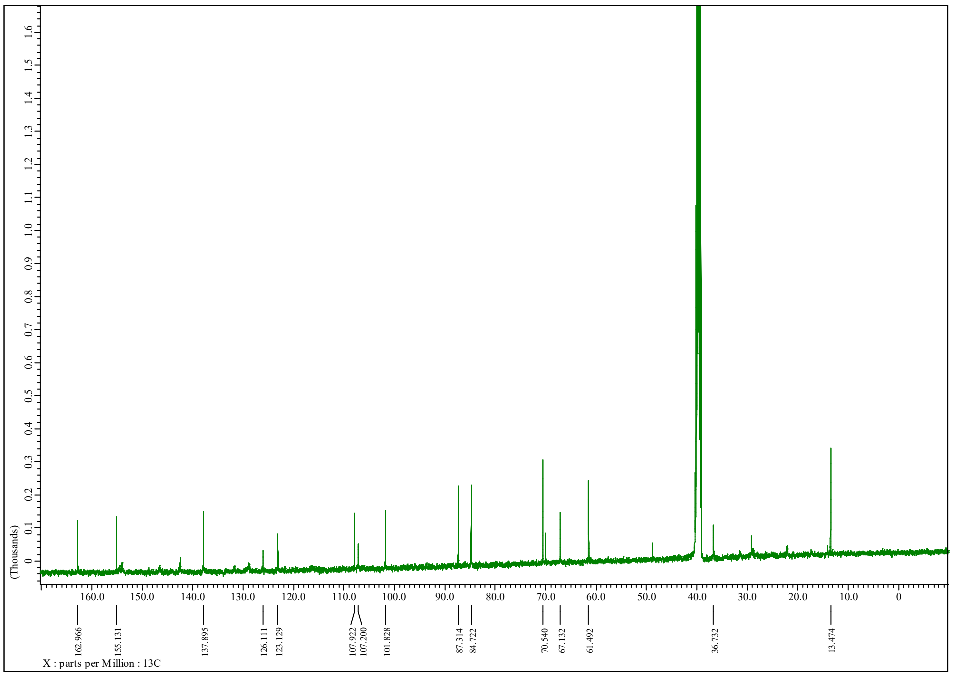

(Thousands)
1.6
1.5
1.4
1.3
1.2
1.1
1.0
0.9
0.8
0.7
0.6
0.5
0.4
0.3
0.2
0.1
0
160.0
150.0
140.0
130.0
120.0
110.0
100.0
90.0
80.0
70.0
60.0
50.0
40.0
30.0
20.0
10.0
0
162.966
155.131
137.895
126.111
123.129
107.922
107.200
101.828
87.314
84.722
70.540
67.132
61.492
36.732
13.474
X : parts per Million : 13C

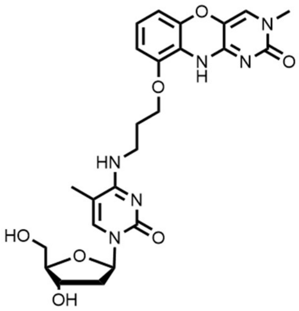

O
N
O
N
H
N
O
HN
N
O
HO
O
N
OH

Compound 2

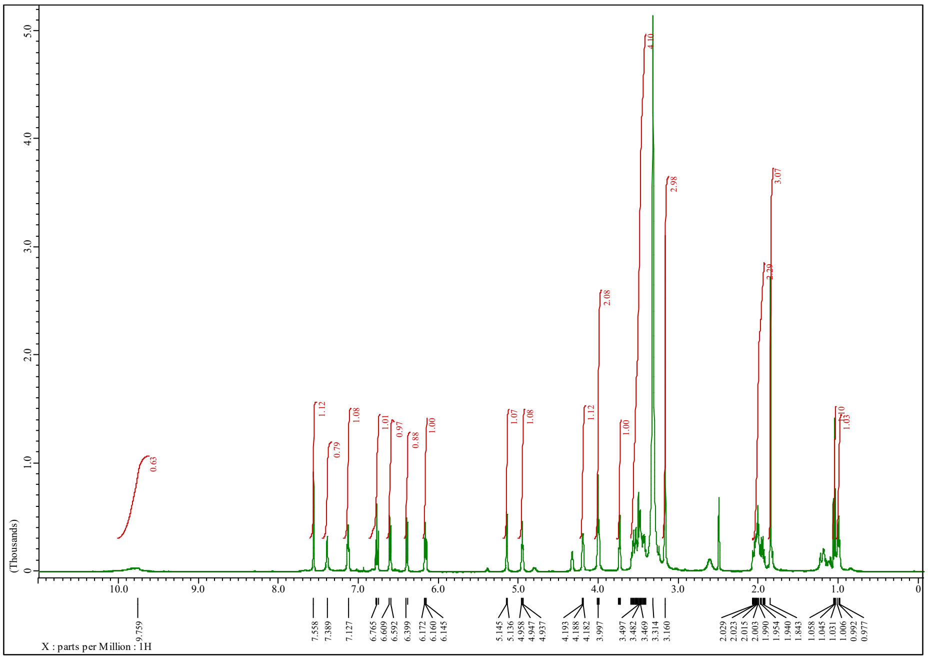

(Thousands)
5.0
4.0
3.0
2.0
1.0
0
0.63
1.12
0.79
1.08
1.01
0.97
0.88
1.00
1.07
1.08
1.12
2.08
1.00
4.10
2.98
2.20
3.07
1.10
1.03
10.0
9.0
8.0
7.0
6.0
5.0
4.0
3.0
2.0
1.0
0
9.759
7.558
7.389
7.127
6.765
6.609
6.592
6.399
6.172
6.160
6.145
5.145
5.136
4.958
4.947
4.937
4.193
4.188
4.182
3.997
3.497
3.482
3.469
3.314
3.160
2.029
2.023
2.015
2.003
1.990
1.954
1.940
1.843
1.058
1.045
1.031
1.006
0.992
0.977
X : parts per Million : 1H

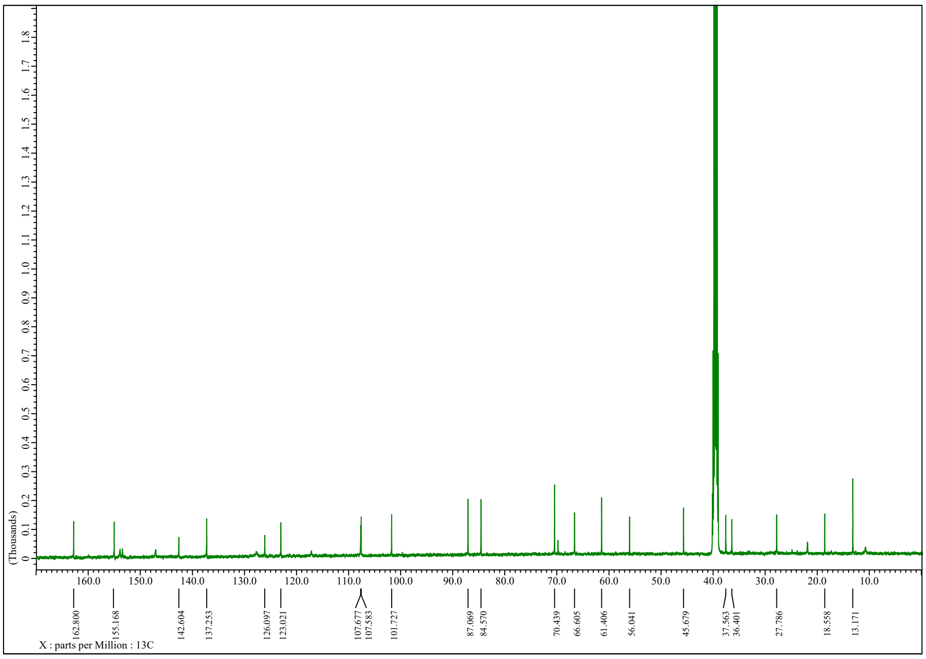

(Thousands)
1.8
1.7
1.6
1.5
1.4
1.3
1.2
1.1
1.0
0.9
0.8
0.7
0.6
0.5
0.4
0.3
0.2
0.1
0
160.0
150.0
140.0
130.0
120.0
110.0
100.0
90.0
80.0
70.0
60.0
50.0
40.0
30.0
20.0
10.0
162.800
155.168
142.604
137.253
126.097
123.021
107.677
107.583
101.727
87.069
84.570
70.439
66.605
61.406
56.041
45.679
37.563
36.401
27.786
18.558
13.171
X : parts per Million : 13C

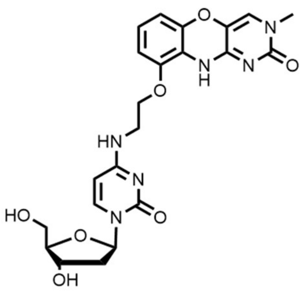
O
N
N
H
O
HN
N
N
O
HO
O
OH

Compound 3

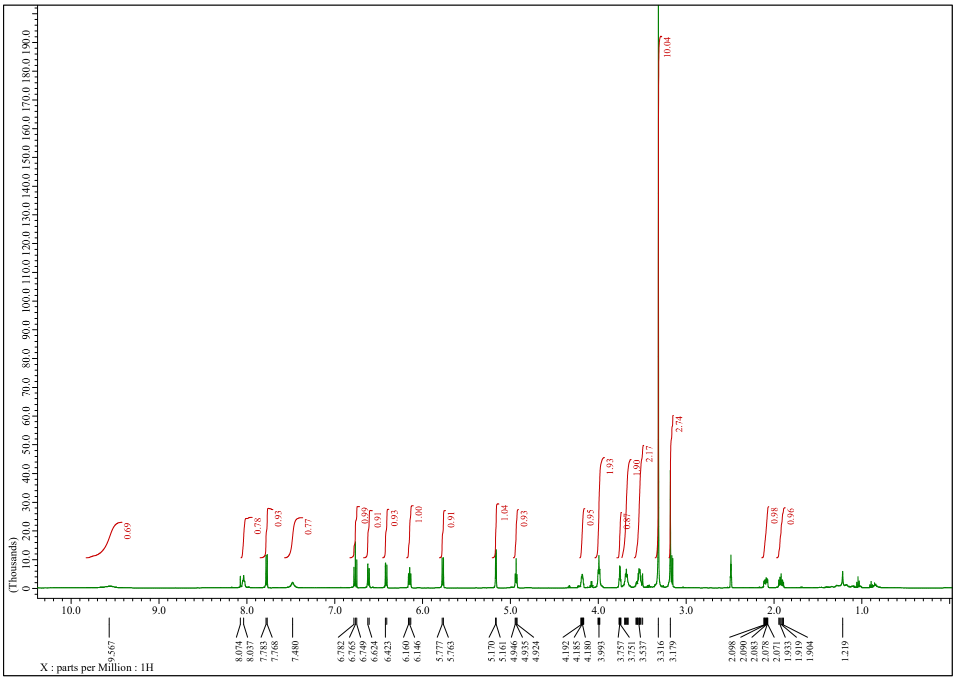
(Thousands)
X : parts per Million : 1H
9.567
8.074
8.037
7.783
7.768
7.480
6.782
6.765
6.749
6.624
6.423
6.160
6.146
5.777
5.763
5.170
5.161
4.946
4.935
4.924
4.192
4.185
4.180
3.993
3.757
3.751
3.537
3.316
3.179
2.098
2.090
2.083
2.078
2.071
1.933
1.919
1.904
1.219

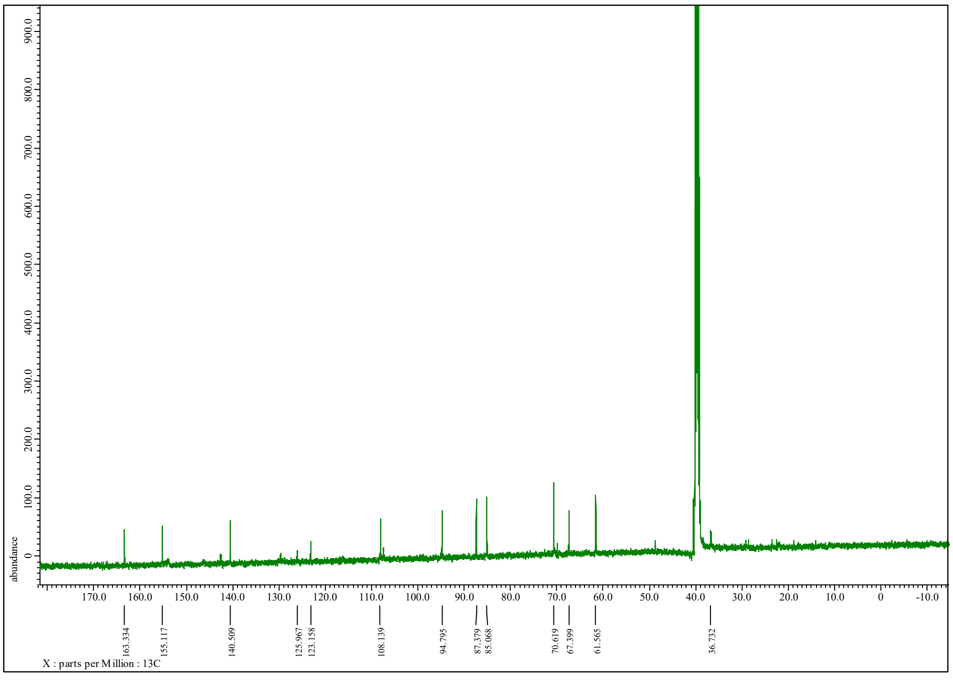
abundance
X : parts per Million : 13C
163.334
155.117
140.509
125.967
123.158
108.139
94.795
87.379
85.068
70.619
67.399
61.565
36.732

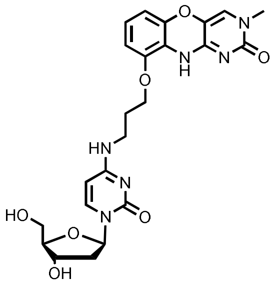

HO
OH
O
N
N
O
HN
O
N
H
N
O
N
O

Compound 4

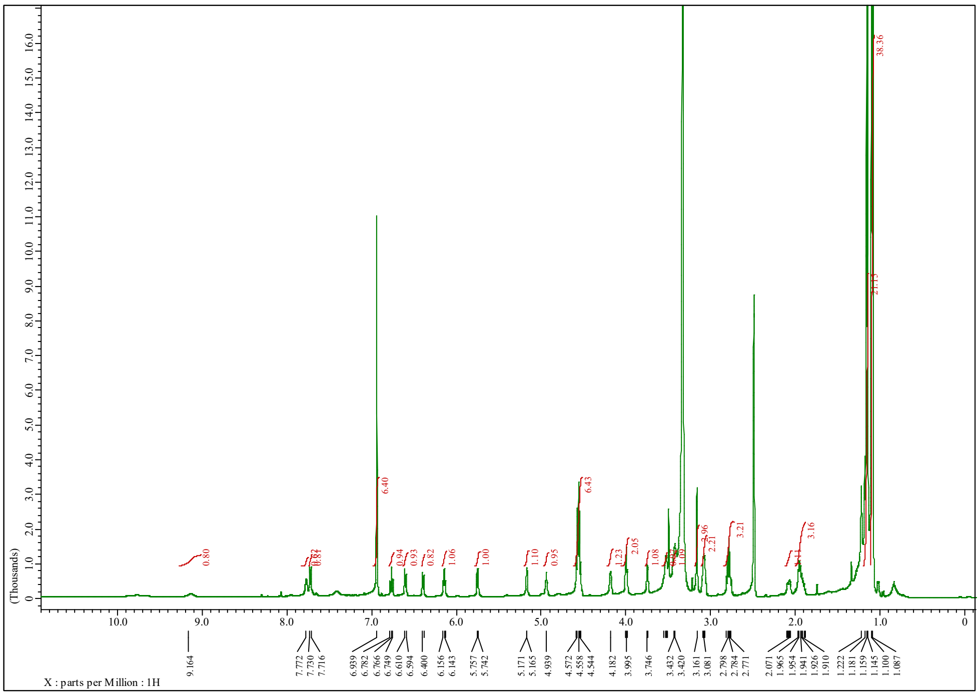

(Thousands)
X : parts per Million : 1H
9.164
7.772 7.730 7.716
6.939 6.782 6.766 6.749 6.610 6.594
6.400
6.156 6.143
5.757 5.742
5.171 5.165
4.939
4.572 4.558 4.544
4.182
3.995
3.746
3.432 3.420
3.161 3.081
2.798 2.784 2.771
2.071 1.965 1.954 1.941 1.926 1.910
1.222 1.181 1.159 1.145 1.100 1.087

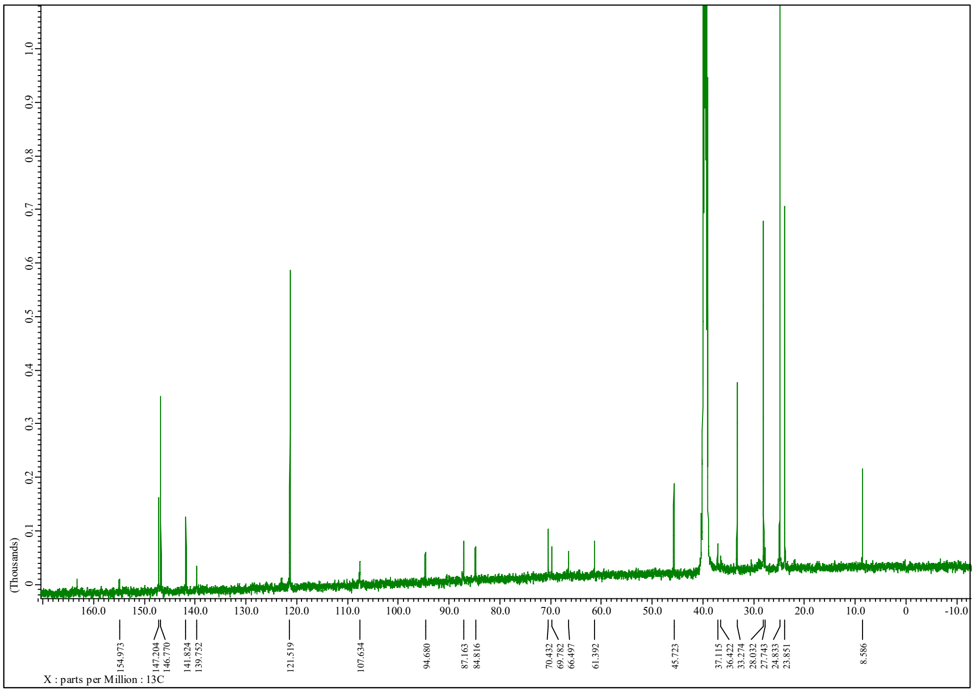

(Thousands)
X : parts per Million : 13C
154.973
147.204 146.770
141.824 139.752
121.519
107.634
94.680
87.163 84.816
70.432 69.782 66.497
61.392
45.723
37.115 36.422 33.274 28.032 27.743 24.833 23.851
8.586

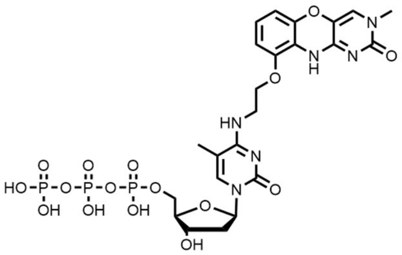

HO
OH
OH
OH
OH
HN
NH
O
N

Compound 17

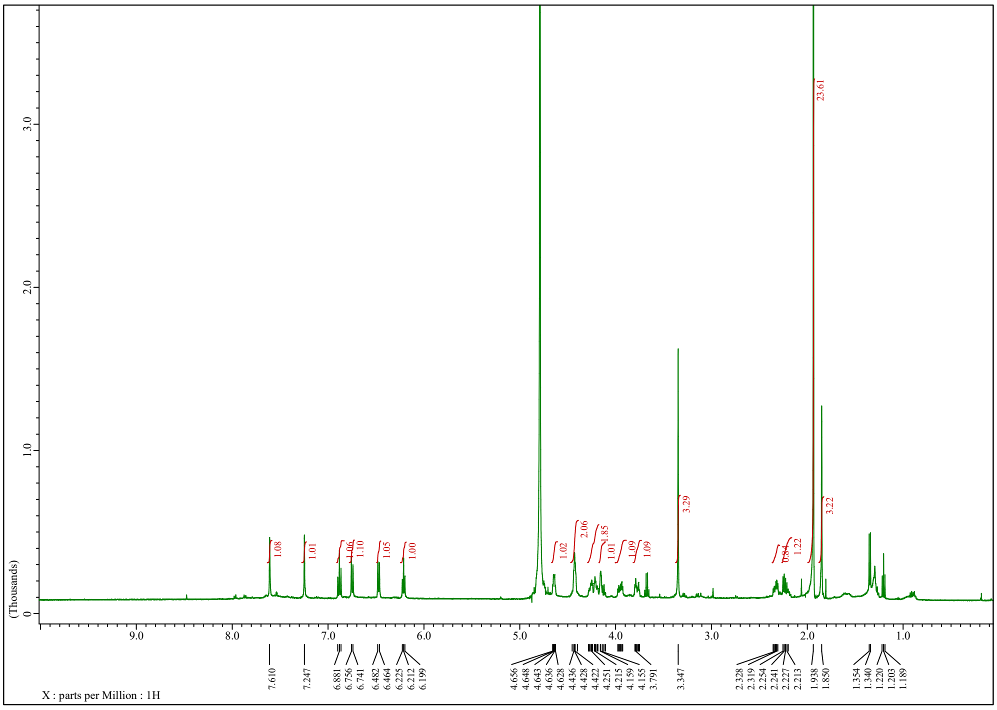

(Thousands)
X : parts per Million : 1H
7.610
7.247
3.347
1.938
1.850

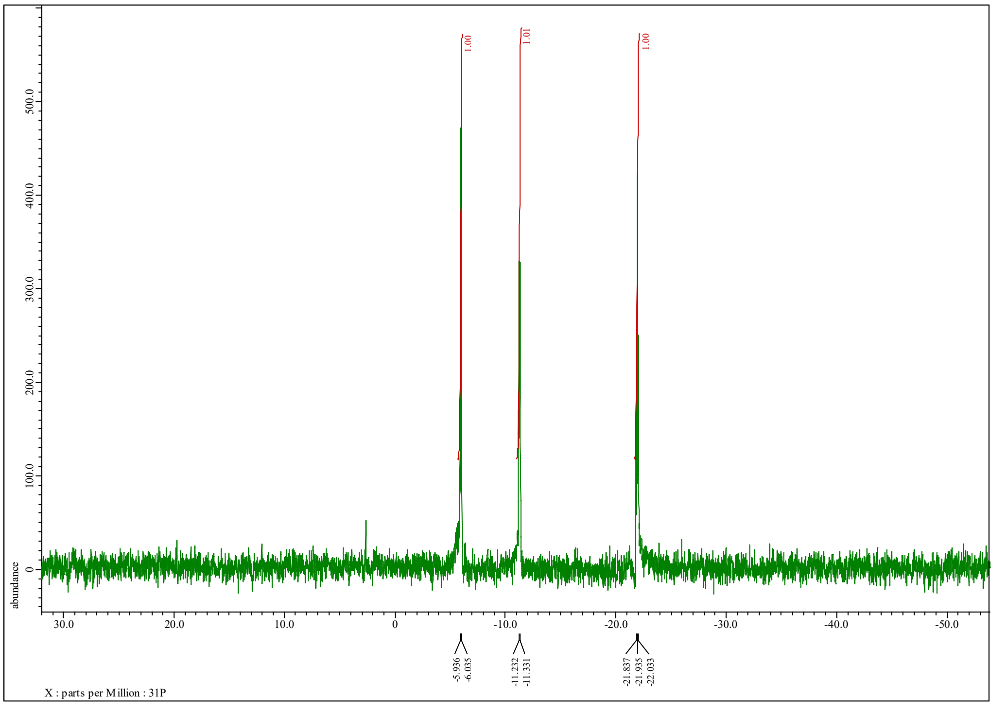

abundance
X : parts per Million : 31P
-5.936
-6.035
-11.232
-11.331
-21.837
-21.935
-22.033

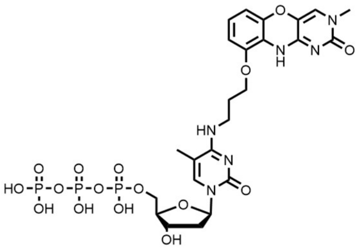
HO
OH
OH
OH
OH
HN
N
O
H

Compound 18

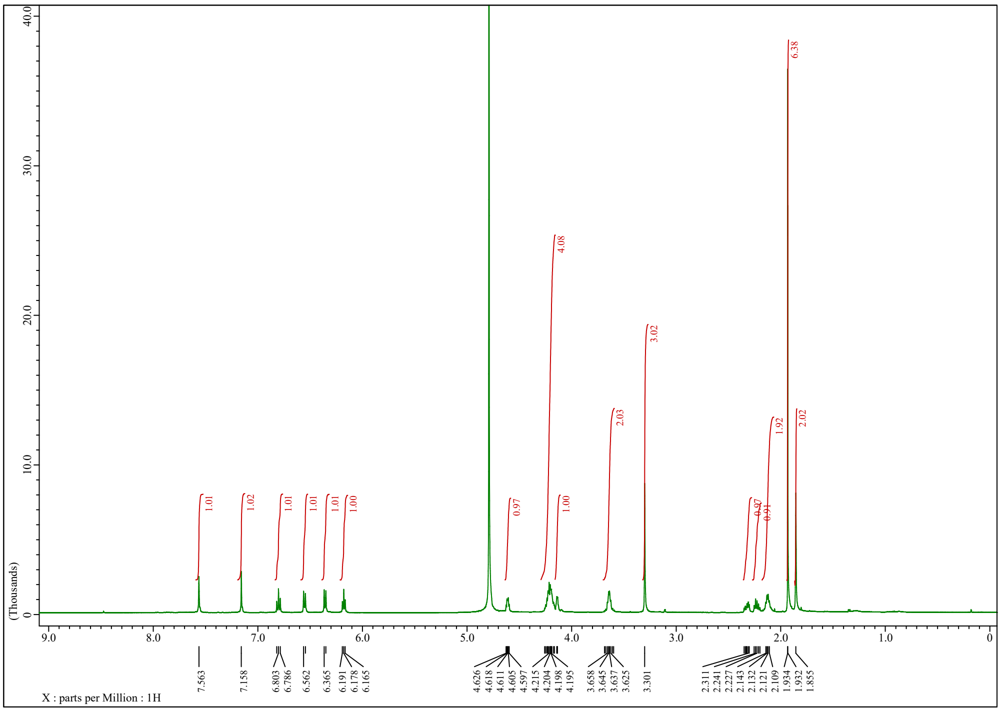
(Thousands)
X : parts per Million : 1H
7.563
7.158
6.803
6.786
6.562
6.365
6.191
6.178
6.165
4.626
4.618
4.611
4.605
4.597
4.215
4.204
4.198
4.195
3.658
3.645
3.637
3.625
3.301
2.311
2.241
2.227
2.143
2.132
2.121
2.109
1.934
1.932
1.855

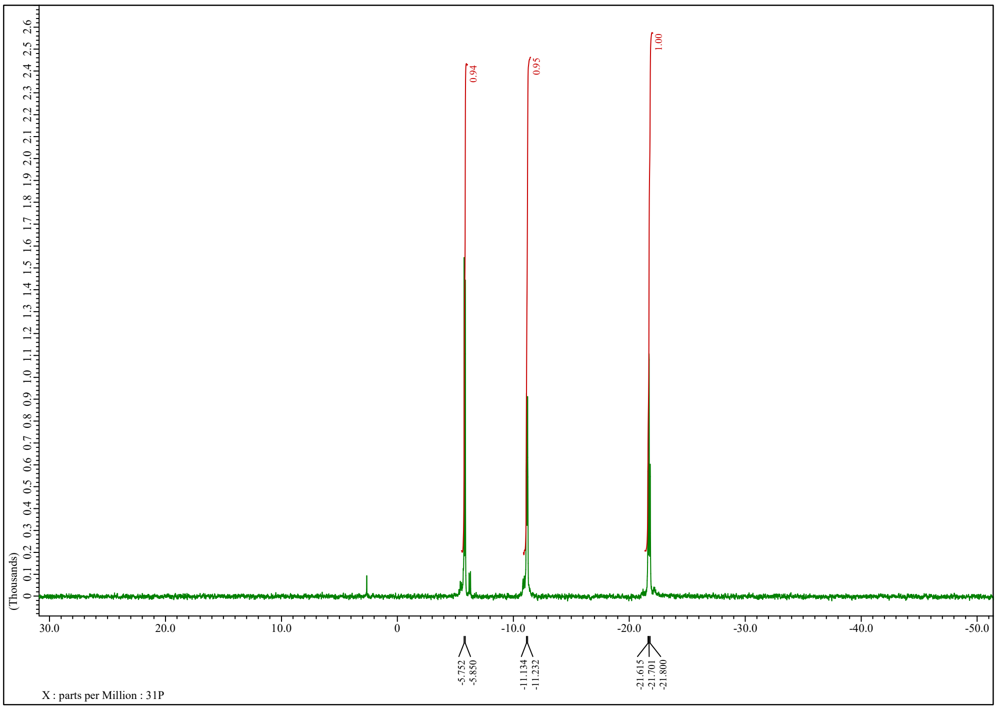
(Thousands)
X : parts per Million : 31P
-5.752
-5.850
-11.134
-11.232
-21.615
-21.701
-21.800

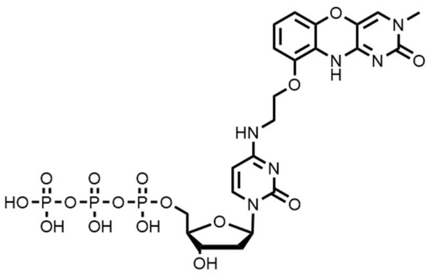

Compound 19

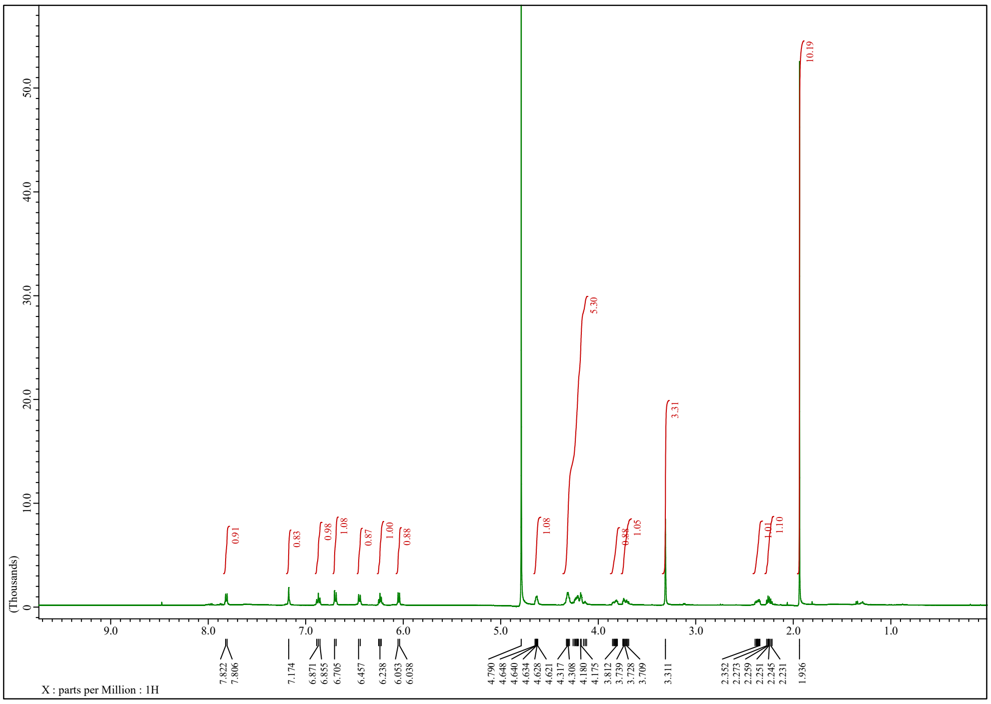

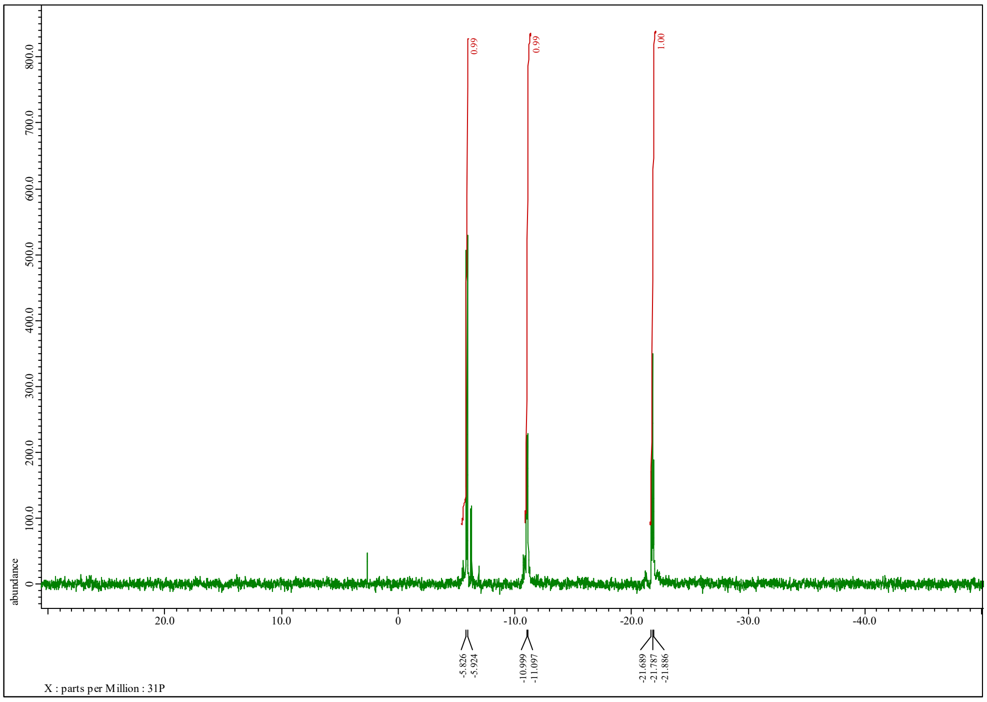

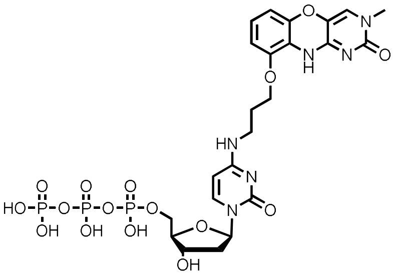

Compound 20

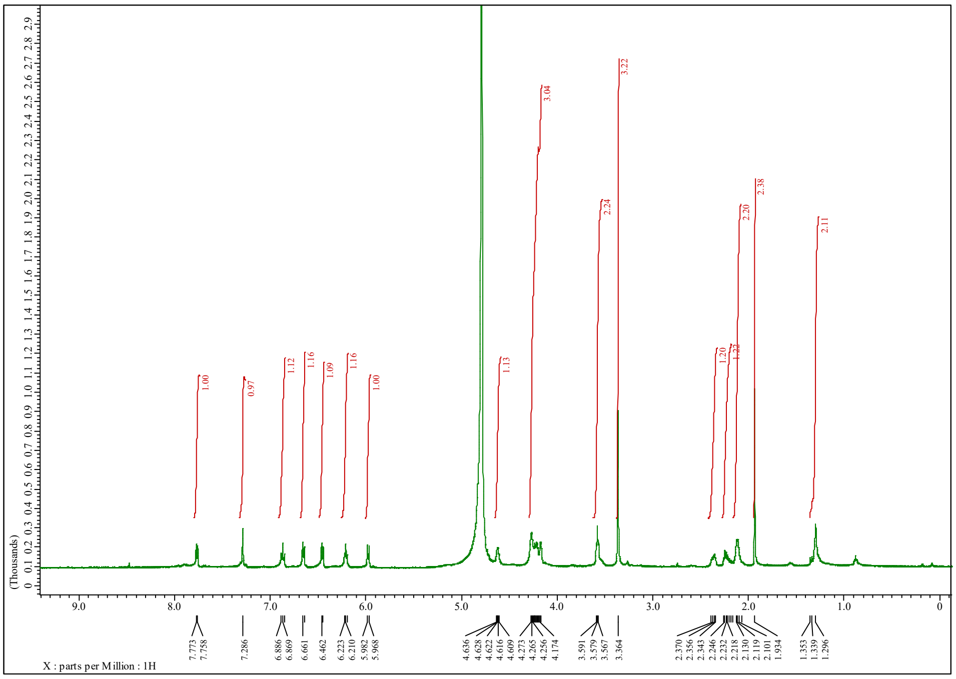

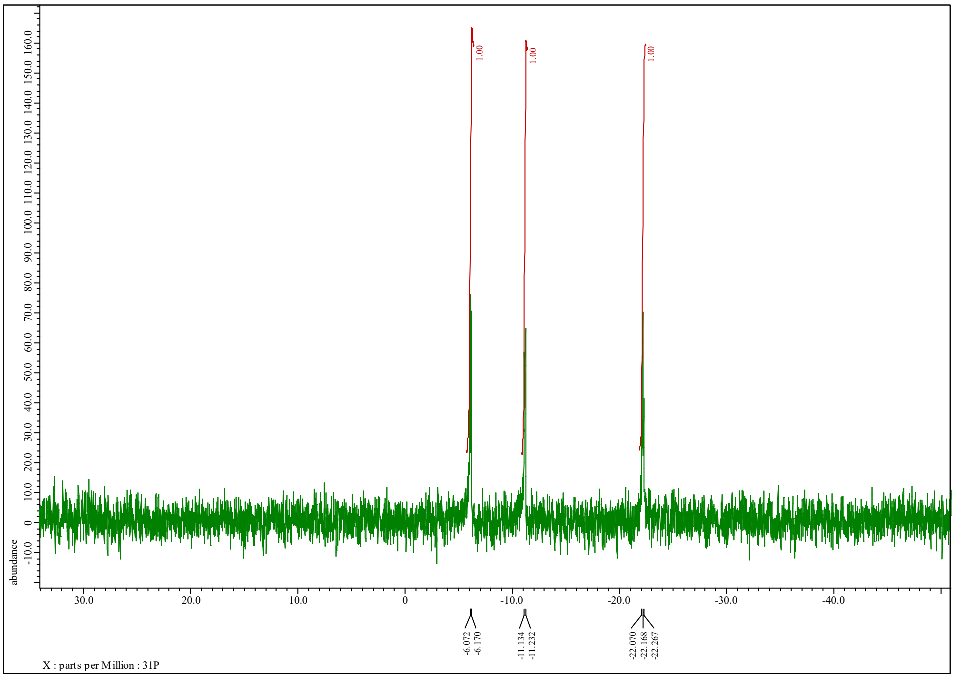

Supplement: Supplementary file 1 [file molecules-29-02270-s001.zip › molecules-2992705-supplementary.pdf]
